# Cranial evolution in the extinct Rodrigues Island owl *Otus murivorus* (Strigidae), associated with unexpected ecological adaptations

**DOI:** 10.1038/s41598-020-69868-1

**Published:** 2020-08-20

**Authors:** Anaïs Duhamel, Julian P. Hume, Pauline Guenser, Céline Salaviale, Antoine Louchart

**Affiliations:** 1 Univ Lyon, Univ Lyon 1, ENSL, CNRS, LGL-TPE, 69622 Villeurbanne, France; 2 Bird Group, Department of Life Sciences, Natural History Museum, Tring, Herts, HP23 6AP UK; 3 Univ Lyon, Université Claude Bernard Lyon 1, CNRS, ENTPE, UMR 5023 LEHNA, 69622 Villeurbanne, France

**Keywords:** Ecology, Evolution, Zoology, Ecology

## Abstract

Island birds that were victims of anthropic extinctions were often more specialist species, having evolved their most distinctive features in isolation, making the study of fossil insular birds most interesting. Here we studied a fossil cranium of the ‘giant’ extinct scops owl *Otus murivorus* from Rodrigues Island (Mascarene Islands, southwestern Indian Ocean), to determine any potential unique characters. The fossil and extant strigids were imaged through X-ray microtomography, providing 3D views of external and internal (endocast, inner ear) cranial structures. Geometric morphometrics and analyses of traditional measurements yielded new information about the Rodrigues owl’s evolution and ecology. *Otus murivorus* exhibits a 2-tier “lag behind” phenomenon for cranium and brain evolution, both being proportionately small relative to increased body size. It also had a much more developed olfactory bulb than congeners, indicating an unexpectedly developed olfactory sense, suggesting a partial food scavenging habit. In addition, *O. murivorus* had the eyes placed more laterally than *O. sunia*, the species from which it was derived, probably a side effect of a small brain; rather terrestrial habits; probably relatively fearless behavior; and a less vertical posture (head less upright) than other owls (this in part an allometric effect of size increase). These evolutionary features, added to gigantism and wing reduction, make the extinct Rodrigues owl’s evolution remarkable, and with multiple causes.

## Introduction

Avian extinctions on islands, because of human encroachment, have been considerable and widespread^1-3^, including those between the seventeenth and nineteenth centuries on Indian Ocean islands^4,5^. These extinct species are not a random sample of insular endemics, but those that tend to be most vulnerable, e.g., having evolved flightlessness, were more terrestrial, or in general considered naive due to the absence of mammalian predators. Therefore, extinct island birds represent extremes of evolutionary trajectories, and this includes island owls^6^, making the anatomical study of these extinct bird species most interesting in terms of insular evolution.

A recently extinct owl (eighteenth century) endemic of Rodrigues Island (Mascarene Islands, southwestern Indian Ocean), *Otus murivorus*, was formerly placed in its own genus, *Mascarenotus*^7^, but a study based on ancient DNA found it to be derived from the much smaller continental Oriental scops owl *Otus sunia* lineage; hence its referral to the genus *Otus*^8^. This insular scops owl had evolved gigantism, becoming twice as large and four times heavier than its continental ancestor, and also had a slightly reduced wing length^8^, all characteristics unseen in extant island owls but observed in some extinct ones, including those on the other Mascarene Islands^8,9^.

**Figure 1:**
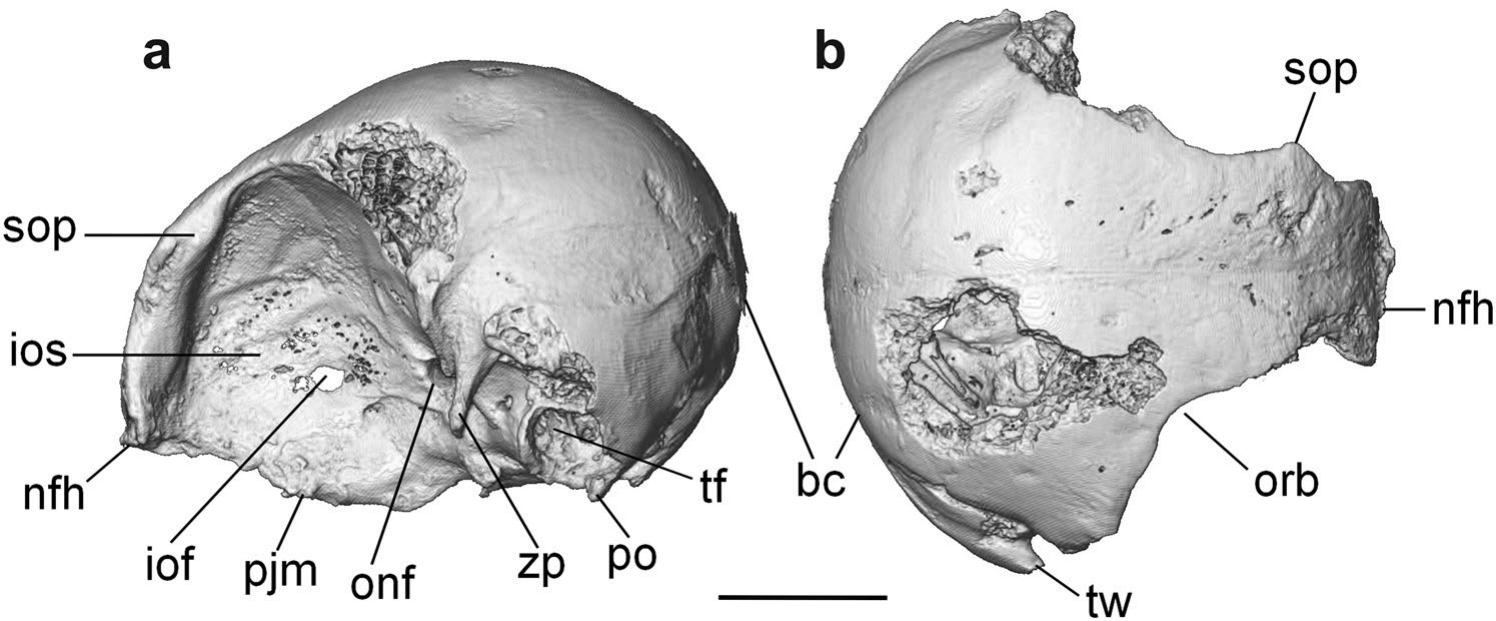
3D reconstruction of *O. murivorus* cranium in lateral left (**a**) and dorsal (**b**) views. bc, braincase; iof, interorbital fenestra; ios, interorbital septum; nfh, naso-frontal hinge; onf, orbital nerve foramen; orb, orbit; pjm, palatines juction with mesethmoid; po, paroccipital process; sop, supraorbital process; tf, tympanic fossa; tw, tympanic wing; zp, zygomatic process. Scale bar, 10 mm.

Preliminary observations suggested a relatively small head, with rather laterally placed and possibly smaller eye orbits in *O. murivorus* than in continental scops owls^8^. The aim of this study is to understand the evolution and the paleoecology of *O. murivorus* in an insular context through its skull’s characteristics. To do so, cranial characteristics were mapped and quantified in *O. murivorus* (Fig. 1), including the evolution of cranial shape using geometric morphometrics (landmarks and sliding landmarks analyses) together with a sample of extant owls, including *O. sunia*, and also comprising various species exhibiting a range of morphological adaptations due to several ecological factors (e.g., diurnality, terrestriality, sedentarity, kind of diet) as well as different body sizes, amongst others. Furthermore, the analysis of avian cranial and endocranial characteristics (endocast, inner ear) in the Rodrigues owl allows observation and measurement of morphological characters known to be associated with particular ecological and behavioural traits, as well as relative development of the different senses, especially those in owls^10-18^. Results are finally discussed in terms of paleoecology and evolution of the Rodrigues owl, in relation to absence of predators and reduction of interspecific competition, with fewer taxa than on the continent, including raptorial birds^6^, the owl being the top predator of its oceanic island^9^.

## Results

### Geometric morphometrics

The STATIS compromise (Supplementary Fig. 1) reveals that *Athene noctua* has a more prominent load than others in structuring the results (correlation circle with data sets), because of the particular lateral, narrow extensions of the supra-orbital processes in this species, absent in the 10 other crania used in this study. The PC1s of dorsal and lateral analyses group together, as do their PC2s (correlation circle with PCA axes), showing that these two analyses summarize the same information (see Supplementary Figs. 2 and 3).

Among PCs, LAT1 summarizes most of the deformation across all species except *A. noctua*, whereas DORS1 summarizes better (as a single PC) deformation between *O. sunia* and its descendent *O. murivorus* (Supplementary Fig. 4). Deformation from *O. sunia* to *O. murivorus* is also visible (shared between PC1 and PC2) in lateral view (Supplementary Fig. 4). In lateral view analysis, PC1 explains 33.5%, and PC2 22.7%, of the total variance. In dorsal view analysis, PC1 explains 53.4%, and PC2 27.1%, of the total variance. In both dorsal and lateral analyses, the broken stick model indicates that PC1 and PC2 are sufficiently informative.

The main deformation across the species (except *A. noctua* and its supra-orbital processes extensions) is correlated with body size, with small owls near one end, and large owls (*Bubo*) near the opposite end, along LAT1. This deformation, from large to small owls, primarily concerns the following: braincase expands proportionately in length relative to orbits, which become more frontal; the basicranium shifts rostrally; and the frontal region changes from flat and extended rostrally, to round and shifted toward the basicranium, giving a round forehead to small owls (Supplementary Figs. 2, 3). Deformation between *O. sunia* and *O. murivorus*, on the other hand, partly differs and as visible along DORS1 it consists of slight narrowing of braincase and widening of frontal region (interorbital). As visible in lateral aspect, deformation between *O. sunia* and *O. murivorus* consists of relatively slight lowering of the dorsal curve of the braincase, more lateral shifting of the caudal limit of orbit, and caudal shifting of the basicranium and ventral edge of interorbital septum.

### Analysis of traditional measurements

The PCA analysis of all measurement data (except angles), transformed using GMs, yields a rather equilibrated distribution of specimens and variables across the four quadrants. The broken-stick model showed that only PC1 and PC2 bore significant information (Supplementary Fig. 5). PC1 accounts for 47.8%, and PC2 27.9%, of the total variance (hence altogether 75.7%) (PCA statistics are in Supplementary Tables 1–3). *Otus murivorus* groups with *B. cinerascens* and *B. zeylonensis* in the same quadrant (Supplementary Fig. 6A).

The specimens scores along PC1 show no correlation to their geometric means (Supplementary Fig. 7, r^2^ = 0.025). This implies that the part of variance expressed by PC1 is non-allometric in general, which includes the fossil. The results will therefore be interpretable in terms of causes other than allometric scaling, i.e., adaptation, and/or correlated evolution of adaptive with non-adaptive trait(s) ^19^.

The correlation circle (Supplementary Fig. 6B) shows the contribution of the variables to the morphological variation and so the factors affecting *O. murivorus* location in the morphospace. The loadings of variables on PC1 and PC2 (Supplementary Fig. 6B, Supplementary Table 3) help make a hierarchy among the factors affecting *O. murivorus*. Compared to *O. sunia*, *O. murivorus* is rather distant (Supplementary Fig. 6A), and exhibits relative cranium thickening, longer olfactory bulb (OB), and wider frontal (interorbital) region (and larger body size), among the more prominent variables. Furthermore, *O. murivorus* exhibits a relative reduction of the wulst (W), as well as differences in global brain (Br) dimensions (brain volume, surface, length, width), foramen magnum size, and the lengths of semi-circular canals (SCs). A few variables show too small loadings on PC1 and PC2 (< 0.1) to be interpretable from PCA alone (Supplementary Fig. 6B, Supplementary Table 3). They are further assessed using other analyses so as to determine which ones are really insignificant taken individually. The more prominent variables (loadings > 0.1) are all considered in detail.

Based on measurements and observations (Supplementary Table 4), bivariate or trivariate analyses and boxplot analyses help characterize *O. murivorus* (see below). Within each category (cranial parts; endocranial parts; measures of angles), the more contrasted features in *O. murivorus*, as they appear in the PCA, are here listed first.

### Cranial parts

#### Cranium relative size

**Figure 2:**
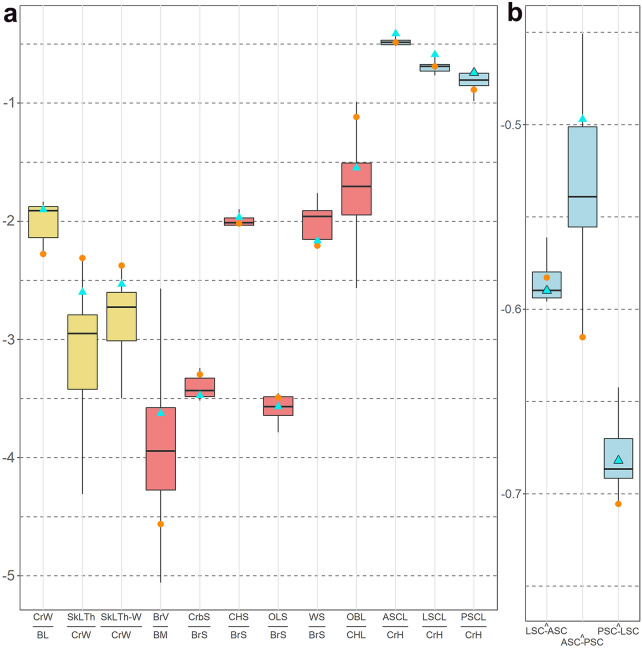
Boxplots showing the position of *O. murivorus* (orange circles) relative to other strigids including *O. sunia* (green–blue triangles), according to a series of ratios (**a**) and angles (**b**) (from data in Supplementary Table 4), expressed as LOG values. The boxes contain the median, the lower and upper hinges correspond to the first and third quartiles. In yellow, cranial ratios; in light red, brain ratios; in blue, inner ear ratios and angles. BL, body length; BM, body mass; BrS, brain surface; BrV, brain volume; CrbS, cerebellum surface; CHL, cerebral hemisphere length; CHS, cerebral hemisphere surface; CrH, cranium height; CrW, cranium width; OBL, olfactory bulb length; OLS, optic lobe surface; SkLTh, skull lateral thickness (at cerebral hemisphere level); SkLTh-W, skull lateral thickness at wulst level; WS, wulst surface. Semi-circular canals abbreviations as in (Fig. 6, Supplementary Fig. 9).

The cranium of *O. murivorus* appears smaller than in all the other strigids studied here, relative to body size (Fig. 2, Supplementary Fig. 8). *O. murivorus* stands significantly and well apart from the allometric trend, being the species with the relatively smallest cranium of the dataset. *O. murivorus* also exhibits cranium size reduction compared with *O. sunia* (Fig. 2, Supplementary Fig. 8). There is an allometric trend of proportionately smaller crania in larger owls (cf. slope in Supplementary Fig. 8), but the noticeable decrease in relative cranium size from *O. sunia* to *O. murivorus* largely exceeds that expected in view of the body size increase of the latter.

#### Relative thickness of cranium bone wall

**Figure 3:**
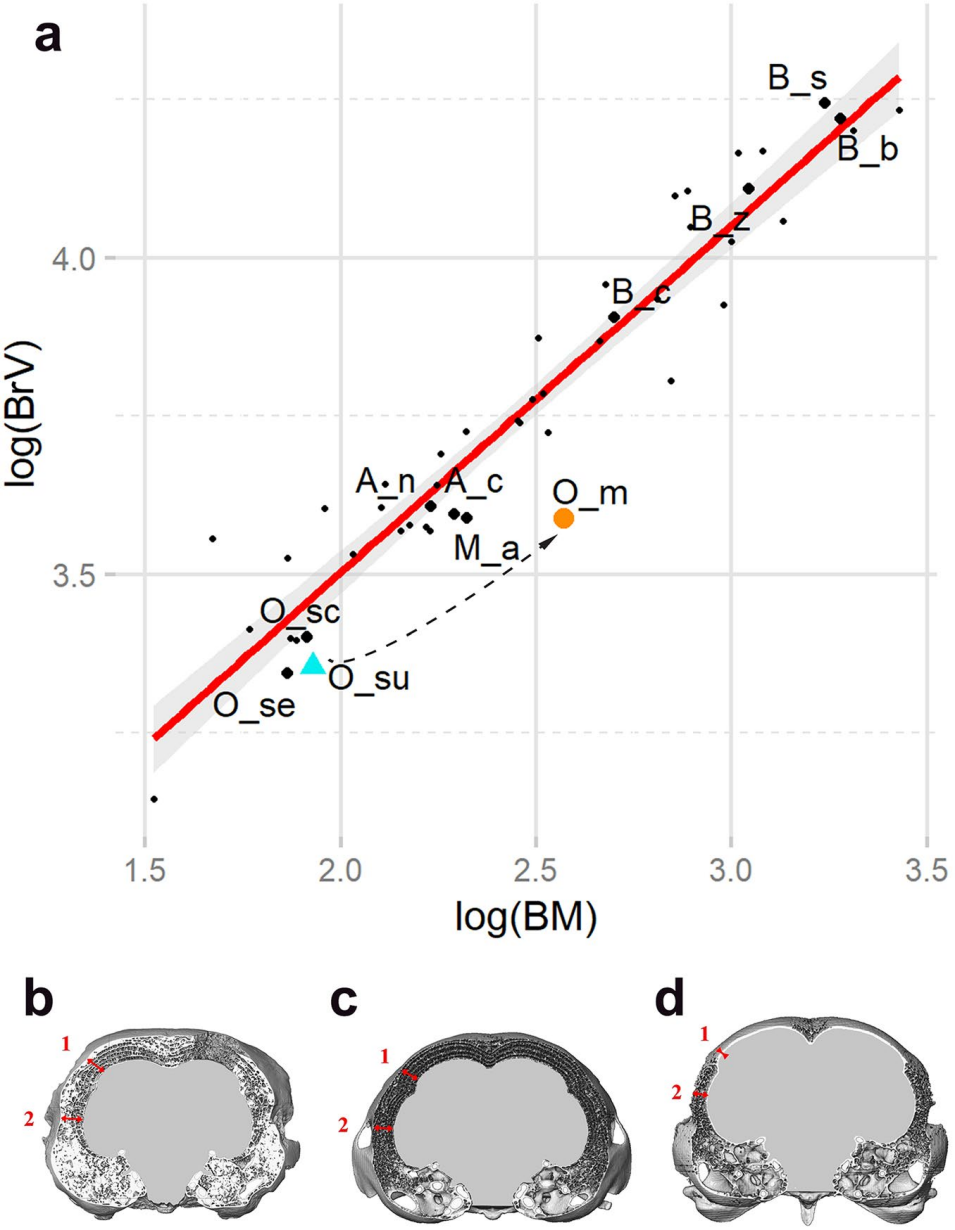
(**a**) Scatter plot of LOG brain volume to LOG body mass (present study data together with published strigid data^21^). *O. murivorus* (orange circle) exhibits the lowest ratio brain volume/body mass of all owls. Dashed arrow shows the evolutionary trajectory from *O. sunia* (green–blue triangle) to *O. murivorus*, which strongly deviates from the allometric trend (Y/X regression line; r^2^ = 0.91). Slope = 0.55. The translucid grey zone represents a 95% confidence interval around the regression. Dashed arrow symbolizes evolutionary trajectory from *O. sunia* to *O. murivorus*. (**b**)–(**d**), Transverse section of 3 skulls in 3D volume at level of foramen magnum, showing the minimal thickness of cranium wall as measured (red arrows) in the wulst (1) and cerebral hemisphere (2) regions, relative to cranium width. (**b**), *O. murivorus*; (**c**) *O. sunia*; (**d**) *O. scops*. Not to scale.

*Otus murivorus* exhibits a high relative thickness of braincase (Figs. 2, 3B–D). Thicknesses of cranium wall measured at higher and lower level on the sides (wulst and cerebral hemispheres region) yield similarly much higher ratios in *O. murivorus* than in other owls including *O. sunia*. In contrast, some extant species exhibit noticeably thin walls, i.e., *O. scops, B. scandiacus*, and *B. bubo* (wulst region only, not cerebral hemisphere region, for the latter species) (Supplementary Table 4).

#### Width of frontal region

Interorbital width, as also appears on PCA results, is higher in *O. murivorus* than in other owls (relative to cranium dimensions); next is *B. zeylonensis* (Supplementary Table 4).

#### Optic and trigeminal nerves foramina

There is no modification in the relative size of optic nerve foramen (visual sense) in *O. murivorus* compared with *O. sunia* and the other owls used in this study (Supplementary Table 4). The relative size of the maxillomandibular foramen for the trigeminal nerve V_2-3_ (tactile sense)^20^ is low in *O. murivorus* compared with the other strigids, and slightly lower than in *O. sunia* (Supplementary Table 4).

### Endocranial parts

#### Brain volume

In *O. murivorus*, the endocranial volume, relative to body size, is markedly lower than in any other owl; this is visible on a scatter plot of LOG brain volume to LOG body mass with a diversity of strigid owls (Fig. 3A) and on boxplots (Fig. 2). *Otus murivorus* is significantly well below the values of the small-brained extant strigids, and attests to an important relative reduction compared with *O. sunia*. There is an allometric trend of proportionately smaller brains in larger owls (cf. slope in Fig. 3). The relative decrease in relative brain volume from *O. sunia* to *O. murivorus* largely exceeds that expected in view of the body size increase of the latter. In addition, the foramen magnum length in *O. murivorus* is relatively slightly reduced compared with *O. sunia* (cf. PCA, and Supplementary Table 4).

#### Olfactory bulb

**Figure 4:**
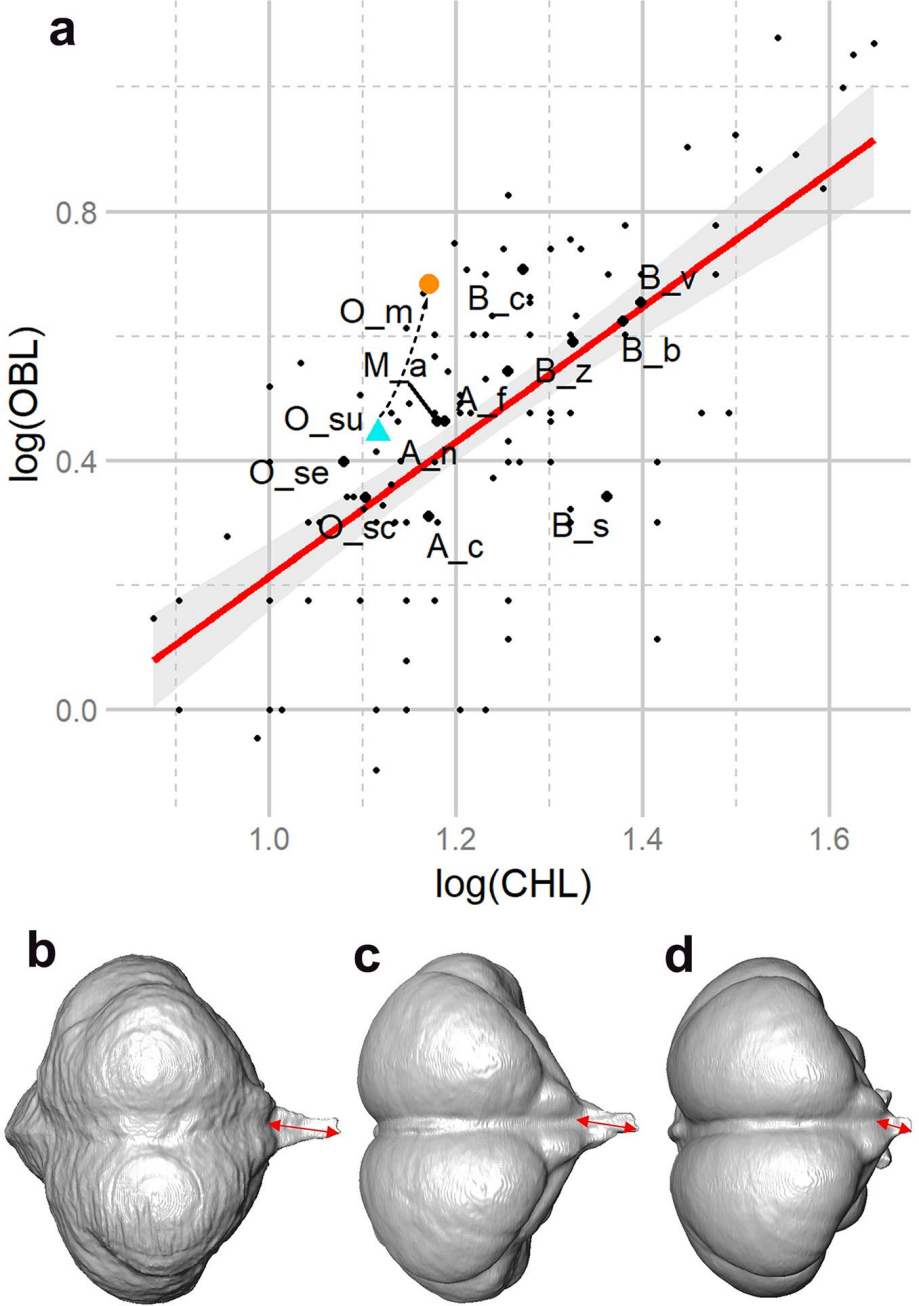
(**a**) Scatter plot of LOG olfactory bulb length (OBL) to LOG cerebral hemisphere length (CHL) (as in Ref.^22^), with the ten extant owls (large black dots; green–blue triangle for *O. sunia*) and the Rodrigues owl *O. murivorus* (orange circle), together with published bird data^18,22^. The Rodrigues owl shows the highest olfactory bulb ratio among strigids, and one of the highests among all birds. Abbreviations additional to this owl’s study (two owls from Ref. ^22^): B.v., *Bubo virginianus*; Asio f., *Asio flammeus.* Linear regression line is Y/X type (r^2^ = 0.48). Slope = 1.1. The translucid grey zone represents a 95% confidence interval around the regression. *Bubo scandiacus* is positioned at the opposite (with lowest olfactory bulb ratio among strigids). Dashed arrow symbolizes evolutionary trajectory from *O. sunia* to *O. murivorus*. (**b**–**d**) Cranial endocasts of *O. murivorus* (**b**), *O. sunia* (**c**) and *A. cunicularia* (**d**). The red arrows show the length of the olfactory bulb (measured as in Ref.^22^). Not to scale.

The relative development of the olfactory bulb in *O. murivorus* (Fig. 4B; here compared with *O. sunia*, Fig. 4C, and *Athene cunicularia*, Fig. 4D) is by far the highest of all strigids (Figs. 2, 4A), and stands among the highest ratio values of all birds^22^. It is comparable to those birds with the greatest olfactory sense (e.g., some procellariiform seabirds and some vultures) ^22^. *Bubo cinerascens* is intermediate between the average values of other owls and that of *O. murivorus*. Compared with *O. sunia*, *O. murivorus* also deviates significantly and strongly from the nearly isometric trend in Fig. 4A.

#### Other endocranial regions

**Figure 5:**
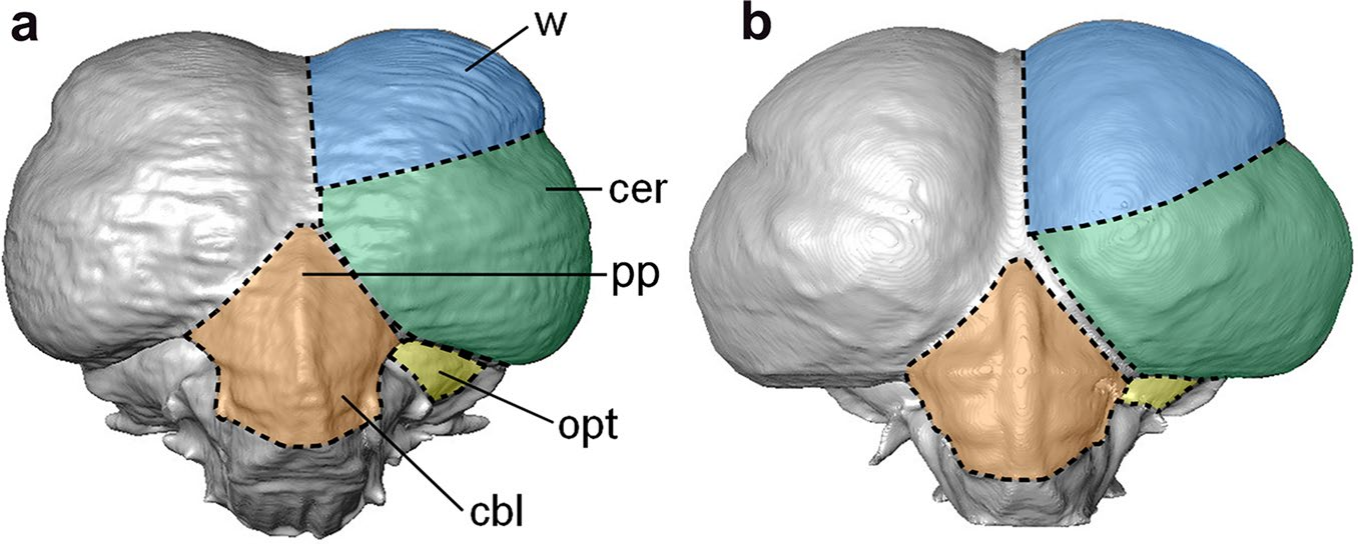
Cranial digital endocasts of *O. murivorus* (**a**) and *O. sunia* (**b**) in caudal view. cbl, cerebellum; cer, cerebral hemisphere; opt, optic lobe; pp, pineal peak; w, wulst. The protuberance of the pineal peak could a priori have been a proxy of diurnal or nocturnal behavior^24,25^, but this detail has proven hardly assessable on the fossil, and moreover, it shows no consistent relation to the more diurnal species examined here. Not to scale.

Ratios of the surface of precise endocranial areas to the total endocast surface add evidence that *O. murivorus* deviates from other strigids in several features (Figs. 2, 5). Proportionately, *O. murivorus* significantly bears a greatly reduced wulst area, as well as a slightly enlarged cerebellum (Crb) compared with all other strigids examined, including *O. sunia*.

Conversely, *O. murivorus* exhibits an optic lobe (OL) surface ratio similar to that of other strigid owls (Fig. 2), and there is no apparent correlation between the surface of the optic lobe and nocturnal or diurnal habits in extant owls (with the more diurnal owls being in the sample *B. scandiacus*, *B. zeylonensis*, *A. cunicularia* and *A. noctua*^23^). Similarly, the cerebral hemisphere (CH) ratio is not significantly different in *O. murivorus* compared with *O. sunia* and other strigids (Fig. 2).

#### Inner ear

**Figure 6:**
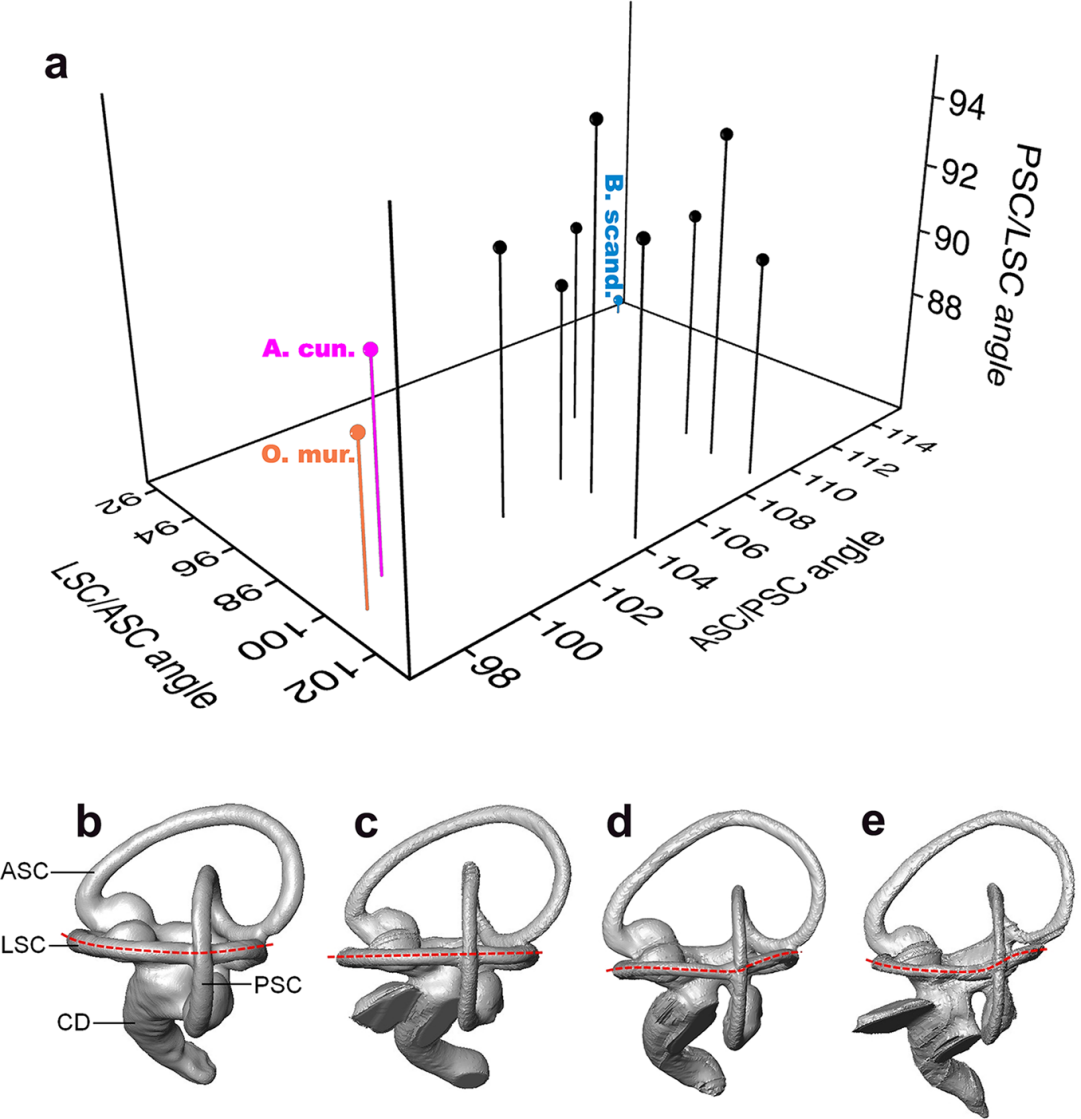
(**a**) 3D scatter plot of the three angles between the three semi-circular canals, in the 11 owls, showing the position of *O. murivorus* (in orange) near *A. cunicularia* (in pink). At the opposite relative to the concentrated positions of the other eight owls is *B. scandiacus* (in blue). (**b**)–(**e**) four 3D view of the inner ear of: (**b**) *O. murivorus*; **c**, *O. sunia*; (**d**) *A. cunicularia*; (**e**) *B. zeylonensis*, showing the sinuosity of LSC (dashed red line). ASC, anterior semi-circular canal; CD, cochlear duct; LSC, lateral semi-circular canal; PSC, posterior semi-circular canal. Not to scale.

Considering the cochlear duct, a ratio of its length (see Supplementary Table 4) to the cranium height ranges from 0.21 (*B. scandiacus*, *B. cinerascens*) to 0.31 (*M. asio*). The difference is slight between *O. murivorus* (0.25) and *O. sunia* (0.28). The lengths of semi-circular canals show more contrasts between species. On a 3D plot, where each axis measures a ratio of one semi-circular canal length to cranium height, the two long-distance migrant scops-owls *O. sunia* and *O. scops* group together with high ratios. A central group contains *O. murivorus* and all remaining species, except *B. zeylonensis*, which exhibits the lowest ratios (Supplementary Fig. 9). *O. murivorus* exhibits a decrease in all semi-circular canals relative lengths compared with *O. sunia* (Fig. 2, Supplementary Fig. 9). The relative thickness of semi-circular canals^16^ varies quite widely among strigids, and it is medium in *O. murivorus*, but proportionately higher than in *O. sunia* (see Supplementary Table 4, Fig. 6B–E). The sinuosity of the lateral semi-circular canal (LSC) ^16^ varies as well. In *O. murivorus*, it is rather medium, hence greater than in *O. sunia*, which exhibits the flattest LSC along with *O. scops* (the two long-distance migrating owls of the sample) (Fig. 6B–E; Supplementary Table 4).

### Measured angles (inner ear and cranium)

#### Inner ear

When plotted in 3D, the three values of angles between semi-circular canals two by two yield three groupings (Fig. 6A). *Otus murivorus* groups with *A. cunicularia*, with medium PSC/LSC angles, high LSC/ASC angles, and the lowest ASC/PSC angles. A group contrasting essentially with moderate to high ASC/PSC angles contains all other owls except *B. scandiacus*. The latter stands at the opposite of *A. cunicularia* and *O. murivorus*, with high ASC/PSC angle, and the lowest PSC/LSC and LSC/ASC angles (Figs. 2, 6A).

#### Orientation of the orbits

In terms of orbital margin convergence, *O. murivorus* exhibits an angle (37°) that attests to a strong decrease compared with *O. sunia* (44.9°). In other words, the orbits are placed more laterally. However, among the owls of the extended sample using data from Menegaz et al. ^26^, *O. murivorus* is in a rather intermediate position, compatible with both nocturnal and diurnal owls, given the wide dispersion of both (Supplementary Fig. 10), and despite there being a slight tendency for lower angles in diurnal species. *Otus murivorus* exhibits an angle more close to the nocturnal mean than to the diurnal mean, but the discrepancy between the two groups is low.

#### Head posture

In terms of posture, as measured as a relative position of the foramen magnum on the cranium, the higher angle in *O. murivorus* compared with *O. sunia* amongst others reflects largely an allometric relation (Supplementary Fig. 11); this angle is higher in large owls (small owls have more ventral foramen magnum). This angle is still slightly high in *O. murivorus*, as it is in *B. zeylonensis* for example.

## Discussion

The main results of the different analyses on the cranium of *O. murivorus* compared with other strigid owls, including with its sister species *O. sunia*, are concordant. They point to: (a) proportionately small cranium, and even smaller brain; (b) relatively long olfactory bulb; (c) enhanced characteristics of inner ear that apparently relate to terrestriality; (d) relative lateralization of eyes; (e) a slightly larger cerebellum and (f) a less vertical head posture. These features are interpreted, in terms of characteristics developed in insular evolution, as a result of a combination of allometric scaling, adaptation to local conditions, and/or correlated evolution sensu Sawada et al. ^19^.

The landmarks/semi-landmarks analyses show that the main deformation across the species is correlated with body size, concurring with Pecsics et al. ^27^. In this context, the deformation observed between *O. sunia* and *O. murivorus* consists of the following morphological modifications. Compared with *O. sunia*, *O. murivorus* evolved the following features in part non-allometrically: orbits more laterally placed, frontal region wider, braincase reduced (width, length, height), and basicranium shifted caudally. This suite of derived traits is original relative to the variation exhibited in the extant species, and explains the offset position of *O. murivorus* in the results of multivariate analyses. In addition, analyses of the traditional measurements taken on cranial and endocranial parts reveal complementary and additional features of *O. murivorus*, as follows, with the most prominent ones discussed first.

### A 2-tier “lag behind” phenomenon for cranium and brain evolution

*Otus murivorus* shows a relatively small endocranium compared with its cranium, and the high thickness of the cranium wall concurs with that observation. In addition, the cranium itself is proportionately reduced in dimensions relative to body size (see Results, cranial parts and endocranial parts-brain volume). The semi-landmarks analyses corroborate this finding, with braincase slightly reduced relative to orbits. In other words, it appears that concomitantly with the doubling in body length from the *O. sunia*-lineage ancestor to *O. murivorus*, the cranium could not grow at the same pace, and the brain increased in size but even more marginally. The relative decrease of cranium size, and in a greater extent of brain volume in *O. murivorus*, are exacerbations of allometric trends in owls (and birds in general), which is in line with a metabolic factor. In *O. murivorus*, limiting the important metabolic cost of developing and maintaining a proportionately large brain is likely to have played a role in the cranium and brain lag behind. A similar finding, concerning only the brain volume, occurred in the giant extinct eagle *Aquila moorei* of New Zealand^28^, interpreted by the authors as a proof of “mismatch between neurological and somatic expansion in an example of evolutionary gigantism” on an island (Ref.^28^: 648). In quite extreme cases of rapid and important somatic size evolution, which is typical on islands^29^, it is structurally more difficult for the brain, being considerably more complex, to undergo such drastic size evolution in a short time^30^. Remarkably, in *O. murivorus* this was accompanied by an intermediate ‘lag behind’ effect of the cranium itself. The body size evolved at a certain speed, the cranium at a lower speed, with an even slower speed for the brain. Therefore, the Rodrigues owl provides a new example, in part similar to another predatory bird, *A. moorei*, of this particular kind of evolutionary pace dissociation. Apparently, a relatively small brain was not a great impediment in the course of evolution in both of these raptorial birds. This characteristic, in an evolutionary trade-off sense, was much more influent in these species than the general opposite and slighter trends for birds to evolve a larger brain on oceanic islands^17^, or for sedentary birds to have a larger brain on average than migratory ones^12^. Interestingly, Sawada et al.^19^found a slightly smaller cranium in the extant *Otus elegans interpositus* in oceanic insular evolution, which suggests that owls do not exactly follow the latter general avian trends described in the above mentioned studies based on samples of extant bird taxa^12,17^.

### A well developed olfactory sense

With one of the largest olfactory bulbs among all birds, *O. murivorus* is likely to have had a highly developed olfactory sense, following the relation established by Bang and Cobb^22^. This ability could probably be associated with scavenging behaviour, since similar values are reached by scavenger birds like vultures, in addition to some procellariiforms^22^. Indeed, partial scavenging habits have already been noticed in owl species like *Bubo cinerascens* (including under *B. (africanus) cinerascens*^23^), in which our results reveal the olfactory bulb is quite well-developed. Moreover, such behaviour among other Rodrigues birds is already documented. For example, the extinct Rodrigues starling *Necropsar rodericanus* scavenged on the super abundant terrestrial tortoise carcasses^31^, as Rodrigues harboured perhaps the densest population of giant tortoises anywhere in the world^32^. The Réunionnais mariner, Julien Tafforet (1725–1726)^31^ described *O. murivorus* as predominantly feeding on small birds, geckos and other reptiles, so presumably a scavenging habit would have been only partial, perhaps seasonal, though more substantial than in *B. cinerascens*.

### A terrestrial species?

The three angles between semi-circular canals are remarkably similar between *O. murivorus* and the Burrowing owl *A. cunicularia*, the most terrestrial owl under study and the only owl in the world to breed in burrows, and both species are distant from the other owls in the morphospace. This particularity of the structure of inner ear in the Burrowing owl has hitherto not been documented. Furthermore, *B. scandiacus* stands alone and well isolated from the central group, opposite to the position of *O. murivorus* and *A. cunicularia*. Indeed, ecomorphologically *B. scandiacus* is the strongest flier among the ten extant species, as it uniquely undertakes long wandering, nomadic flights thousands of kilometers across the wide expenses of its arctic habitats, capacities reflected for example in its long wings^23^. Even though links between inner ear canals shape and angles with flight styles is not obvious across all birds^16^, our findings support a relationship within the Strigidae between semi-circular canals angles and flight capacities, with the position of *O. murivorus* indicating relatively poor flight capacities and probably quite terrestrial habits. The sedentarisation and the reduction of flight capacity in the Rodrigues owl are also suggested^16,33^by the reduction of ASC, LSC and PSC lengths, and by an increase of *O. murivorus*’ LSC sinuosity, compared to *O. sunia* and *O. scops*, which are both long-distance migratory species. This is consistent with the tendency in stronger flying birds to have relatively longer canals^16^. The thickness of the semi-circular canals is undocumented in other birds and hard to interpret^16^, as is their difference between *O. murivorus* and *O. sunia*. Therefore, if further investigations would be welcome to confirm this, the study of the inner ear suggests some level of terrestrialisation and sedentarisation in *O. murivorus*, which is confirmed by the reduction of wings already recorded in this species (as well as the two other extinct Mascarene *Otus* owls)^7,8^.

With such terrestriality, it is possible that *O. murivorus* may have nested on the ground, as did other extinct island owls on the Hawaiian Islands and in New Zealand^32^. It is interesting to note that woodpecker holes are often necessary for scops-owls to nest elsewhere^23^, but as there was no woodpecker on Rodrigues or on the other Mascarene Islands, cavity nest holes were presumably much more difficult to obtain. Furthermore, two species of large, cavity-nesting parrots once occurred on the island^32^, which would have made competition for nesting sites much more severe.

### A less binocular eyesight

Several lines of evidence in *O. murivorus* reveal that it had a less binocular (more lateral) eyesight. The orbital convergence angles, spacing between eyes, wide frontals between orbits (see PCA on measurements, and measured angles), as well as the semi-landmarks analysis are all indicative. In addition, a relatively weakly developed wulst is clearly correlated with less binocularity^13,14^, which is remarkably concordant with the morphology described above. This lateralisation of eyesight in *O. murivorus* compared with the ancestral state visible in *O. sunia*, can be explained by a phenomenon of “correlated evolution”. The frontal position of orbits in owls in general is considered a consequence of an increased encephalization together with the increased space occupied by auditory organs, with owls having altogether high visual and auditory capacities, rather than any direct adaptive causes^26,34^. Therefore, once the constraint of a larger brain was slightly relaxed in *O. murivorus*, the eyes became situated in a more lateral position. This phenomenon of “correlated evolution” would have overpassed in amplitude the binocularisation observed in the oceanic insular owl *O. elegans interpositus* in relation with the absence of predation^19^. The latter study’s main conclusions pointed at a slightly smaller skull, but with eyes positioned slightly more frontally in *O. e. interpositus* compared with other subspecies^19^. Therefore, there is obviously a trade-off between these two trends, frontalisation vs. lateralisation, but there is no clear adaptiveness in the lateralisation of eyes in *O. murivorus*.

### A probably fearless species

The relatively greater development of *O. murivorus*’ cerebellum may be interpreted in terms of flight initiation distance (FID). Such a relation has been identified among birds, albeit slight, and stated by Symonds et al. (Ref.^35^: 6) as: “Although cerebellum size was the most strongly weighted brain component in our analysis, its importance was still weak, and the analysis suggests a negative relationship to FID”. This would suggest that *O. murivorus* had a reduced flight initiation distance; hence the species would have been relatively more fearless than its ancestor and most owls in the present study. This is a classic phenomenon in insular birds that had no native predator until the arrival of humans and their commensal animals^36^. Interestingly, the two *Athene* species, which are the most human-tolerant of the ten extant species^23,37^, also show a rather high cerebellum ratio.

### A less vertical head posture

The relative position of the foramen magnum and the study of landmarks suggest that *O. murivorus* had a less upright head position than its closest congener, but this posture can be mainly explained by its larger size (allometry). Small owls have an upright head position, whereas large owls such as *Bubo* spp. exhibit a more forward-inclined posture with the head positioned more forward. This position, which is slightly exacerbated in *O. murivorus* relative to the allometric trend, is closest to that of *B. zeylonensis*.

### Others

There is no significant evidence of a more diurnal or nocturnal behaviour in *O. murivorus*, neither through PCAs (no close relation within diurnal species), nor through pineal peak, optic nerve foramen, or optic lobe observation. Extended comparisons of the orbital margin convergence angle even suggest a closer position to more nocturnal and crepuscular species, but it is not sufficiently contrasted even among extant species, to be assessed. It is likely that there is no significant change in *O. murivorus* nocturnal vs. diurnal habits, compared with *O. sunia* and most congeners. This would be in agreement with contemporary observations, when Tafforet^31^ described both a crepuscular and nocturnal activity for *O. murivorus*. Concerning auditory capacity, the very slight decrease of cochlear duct length in *O. murivorus* seems to be of little evolutionary significance. Finally, a slight decrease in size of the maxillomandibular foramen for the trigeminal nerve V_2-3_ (tactile sense nerve toward mandible and rostrum) in *O. murivorus*, compared with *O. sunia* (and the lowest value in the strigid sample), is difficult to interpret since no direct explanation elucidates the variation seen among extant species. However, one possibility may relate to the proportionately small beak in *O. murivorus*^8^.

## Conclusion

In summary, the Rodrigues owl evolved a mixture of characteristics in adaptation to an oceanic insular context (terrestriality, reduction of flight, fearlessness) and available prey (olfaction), but also in relation to the direct and indirect effects of insular gigantism in the first place. Direct effects of allometric scaling include change in head posture, whereas indirect effects include a lag behind in both cranium and brain evolution, which adds originality to an already known insular phenomenon of brain lag behind reported in an extinct New-Zealand bird of prey. In *O. murivorus*, the lag behind in brain evolution is interpreted as having induced more laterality in the position of eyes. However, prior to human arrival, neither a relatively small brain, nor correlated reduced binocularity appears to have had any noticeable negative effects on the owl’s existence on pristine Rodrigues.

## Material and methods

### Material and image treatment

The *Otus murivorus* fossil cranium FLMR617 (Fig. 1), from Rodrigues Island^5,38^, and ten crania of extant owls, were imaged through x-ray microtomography at ENS de Lyon (GE Phoenix Nanotom 180 device (platform US8/UMS3444 SFR BioSciences), in order to obtain 3D reconstructions of cranium, endocast and inner ear, as well as virtual sections of cranium. Brain endocast is considered to be an excellent proxy of the underlying brain structure in birds^39^. Avizo Lite 9.0.1 and Meshlab 2016 were used for image treatment and measurements (provided in electronic supplementary material) (Supplementary Figs. 12–16). The extant owls were chosen for covering a large array of behaviours, activity patterns (nocturnal to diurnal), flight capacities and flight styles, diets and sizes (from 73 g and 175 mm length for *O. senegalensis*, to 1900 g and 620 mm length for *B. bubo*, Supplementary Table 4), and include *O. sunia*, the sister species to *O. murivorus*^8^. Osteological characteristics of extant *O. sunia* are confidently considered representative of their direct continental ancestral lineage at 3.5 Ma^8^ (see Supplementary Text). Osteological nomenclature follows^40^, and systematics follows^23^.

### Landmarks and semi-landmarks analyses

Landmarks and semi-landmarks analyses were used to decipher shape modifications in *O. murivorus* in comparison with ancestral *O. sunia*, and also with other strigids in order to detect possible convergences. In dorsal view, 100 semi-landmarks, and in lateral view 4 landmarks and 65 semi-landmarks in two dimensions (Supplementary Fig. 17, Supplementary Table 5; and see Supplementary Text, for justification) were digitised using Tps Dig 2^41^; semi-landmarks were allowed to slide^27^. After Procrustes superimposition using TPS Relw 32^41^, consensus configurations and relative warps were processed and visualised as a Principal Component Analysis (PCA) with PAST 3^42^. In an attempt to assess the contribution of characters caught by both views in the overall morphological variation of the assemblage, a STATIS compromise was built following^43,44^.

### Traditional measurements analyses

A PCA was applied to all measurements taken (Supplementary Table 4, except angles) and weighted with the geometric mean (GM) in order to evaluate the influence of allometry, as well as possible covariation between different variables, their relative contribution to each informative principal component (PC), and so the overall morphological variation. The informative PCs are determined using a broken-stick model provided in the PCA results with PAST 3, following^45^.

The PCAs results were completed by bivariate and trivariate plot analyses, as well as boxplot analyses, derived from traditional measurements taken (Supplementary Table 4).

Detailed methods are provided in the electronic supplementary material.

## Supplementary information

Supplementary file1 (PDF 8122 kb)

## Data Availability

Digital data used in this study are available from the Dryad Digital Repository: https://doi.org/10.5061/dryad.vdncjsxrj.^46^ Otherwise the datasets supporting this article have been uploaded as part of the electronic supplementary material.
