# Supplementary material for: Cranial evolution in the extinct Rodrigues Island owl Otus murivorus (Strigidae), associated with unexpected ecological adaptations
Source: Sci Rep. 2020 Aug 20;10:14019. doi: 10.1038/s41598-020-69868-1 (PMC7441405; doi:10.1038/s41598-020-69868-1)
Supplement: Supplementary file 1 — Supplementary file1 (PDF 8122 kb) [file 41598_2020_69868_MOESM1_ESM.pdf]

Supplementary Information

Cranial evolution in the extinct Rodrigues Island owl *Otus murivorus* (Strigidae), associated with unexpected ecological adaptations

Anaïs Duhamel, Julian P. Hume, Pauline Guenser, Céline Salaviale & Antoine Louchart

This PDF file includes:

Supplementary Material and Methods
Supplementary Figures 1 to 17
Supplementary Tables 1 to 5
References

Supplementary Material and Methods

Material

Extant species

The following specimens of extant Strigidae were used for comparisons and integrated in the analyses: *Otus scops* male (UCBL-FSL-SKT 246.2), *O. sunia* (MNHN-LAC 2000.329), *O. senegalensis* (MNHN-LAC 1997.476), *Megascops asio naevius* (UCBL-FSL-SKL 1856-540), *Bubo scandiacus* male (UCBL-FSL-SKL 2626), *B. bubo* male (UCBL-FSL-SKL, no number, from Ariège), *B. cinerascens* (UCBL-FSL-SKL 453, FSL), *B. zeylonensis* male (MNHN-LAC 1986-05), *Athene noctua glaux* (UCBL-FSL-SKL 568-379), *A. cunicularia* (UCBL-FSL-SKL 656-436).

Institutions abbreviations: FLMR, François Leguat Museum, Rodrigues, Republic of Mauritius; MNHN-LAC, Laboratoire d'Anatomie Comparée, collection of bird skeletons, Muséum National d'Histoire Naturelle, Paris, France; UCBL-FSL-SKL, collection de la Faculté des Sciences de Lyon de l'Université Claude Bernard Lyon 1 (Villeurbanne, France), category extant bird skulls; UCBL-FSL-SKT, collection de la Faculté des Sciences de Lyon de l'Université Claude Bernard Lyon 1 (Villeurbanne, France), category extant bird skeletons.

Selection of these ten particular extant species covers a large array of behaviours, activity patterns (nocturnal to diurnal), flight capacities and flight styles, sizes and diets. In addition, the extant *Otus* includes *O. sunia*, the sister species to *O. murivorus*; the split between the *O. sunia* lineage and that of *O. murivorus* occurred at ca 3.5 Ma[1]. Continental owls do not display significant morphological phyletic changes in such a short time period; in addition, continental species in the genus *Otus* are remarkably homogeneous osteologically, including size among related species. Therefore, osteological characteristics of extant *O. sunia* are confidently considered representative of their direct continental ancestral lineage at 3.5 Ma[1].

Fossil cranium of *O. murivorus*

The cranium studied here (Fig. 1) (FLMR617) was found in 1997 by Nick Arnold and Carl Jones in a cave named Caverne l'Affouche on Rodrigues Island and, along with a large collection of other vertebrate material, given to one of us (JPH; NHM Tring Bird collections, UK) in 1998 to identify and use for future study. It is one of only two known such elements for this species, the other cranium being described by Günther and Newton[2]. The referral of FLMR617 to *O. murivorus* is straightforward. This cranium exhibits the wide, round, rather forward facing orbits of Strigiformes, and differs from tytonid owls in the much more convex and shorter frontal area (in tytonids frontal bones form a straighter (slightly S-shaped), oblique forehead viewed laterally, with a sagittal better marked groove in the midline); wider orbits and relatively wider braincase in dorsal view. Among strigid owls, the fossil cranium agrees well with the group containing scops-owls (*Otus* and allies), and differs from other strigids, in the combination of: regularly round (bulbous) braincase in dorsal view, small supraorbital processes, rather high and steep forehead, amongst others. In dimensions, it agrees with the expected size of *O. murivorus*, in which linear postcranial dimensions are generally twice those of an ordinary *Otus* species such as ancestral-like *O. sunia*[1]. Hence, the cranium dimensions, like those of the other cranium described[2], are roughly 1 ½ times those of *O. sunia*. This slightly lesser size increase in skull compared with body dimensions in *O. murivorus* is analyzed here and discussed, along with other original features. The present fossil cranium therefore corresponds well with *O. murivorus*, and it is known, in addition, that no other owl (Strigiformes) was (and is) present on Rodrigues Island, as is the case on the two other Mascarene Islands, where two endemic *Otus* are now extinct, and each one was the only owl on each island[3]. The *O. murivorus* fossil is hereafter referred to as a cranium, because the term 'skull' refers to the cranium and rostrum combined, but the latter is unavailable.

The specific epithet '*murivorus*' (*murivora*) meaning 'eating mice' was coined by A. Milne-Edwards[4] because the owl was thought to have eaten mice and rats (see the testimony of F. Leguat), but this was possibly the case only after human colonisation, since the native fauna of Rodrigues and

other Mascarene Islands comprised no mammals except micro and macrochiropteran bats. Before the arrival of humans and in the very least, *O. murivorus* fed on birds and reptiles (see main text), but not on mammals.

X-ray microtomography
The 11 specimens (the fossil and 10 modern skulls) were imaged through conventional X-ray microtomography. These CT-scans were performed at Ecole Normale Supérieure de Lyon with a GE Phoenix Nanotom 180 device (platform US8/UMS3444 SFR BioSciences). The following parameters were used for the different specimens (voltage, 100 kV; current, 70 µA). Voxel sizes were as follows: *O. murivorus*, 18 µm; *A. cunicularia*, 23 µm; *A. noctua*, 24.3 µm; *B. bubo*, 32.5 µm; *B. cinerascens*, 32.2 µm; *B. scandiacus*, 30 µm; *B. zeylonensis*, 32.2 µm; *M. asio*, 23 µm; *O. scops*, 18.3 µm; *O. senegalensis*, 19.3 µm; *O. sunia*, 20 µm.

Image treatment and measurements
The virtual volumes and sections were treated and analyzed using Avizo Lite 9.0.1 software. Measurements (linear, angles, volumes) were taken using Avizo (Supplementary Figs. 12-15). Linear measurements taken on the crania are explained in Supplementary Figs 12,13. In addition, Fig. 3B-D (see main text) shows how the two measurements of cranial wall thickness were taken. Linear measurements taken on endocasts are explain in Supplementary Fig. 14. In addition, the measurements used for olfactory bulb ratio[5] are explicited in Fig. 4B-D (see main text) and Supplementary Fig. 16; measurements were taken as in refs. [5,6] (maximal diameter of olfactory bulb relative to the maximal diameter of cerebral hemisphere (CH) of the same side); the latter method has potential shortcomings recalled in refs. [5,6], but no better method is available, and it allows comparison with an extended dataset; furthermore, it gives satisfactory and coherent results again in the present study. Angles measured on crania are explicited in Supplementary Fig. 13. Angles measured between semi-circular canals (SCs) of the inner ear are explicited in Supplementary Fig. 15. Measurements of surfaces of specific areas of the brain (Br) (and total brain surfaces) were taken using Meshlab 2016, the surfaces measured are the colored areas of Supplementary Fig. 16. Orthographic views are always used for observations and treatments (including landmarks and semi-landmarks analyses ; see below), and for all measurements (provided in Supplementary Table 4).

Landmarks and semi-landmarks analyses
Landmarks and semi-landmarks were digitised, using Tps Dig 2[7] in two dimensions in dorsal and in left lateral views, as two separate analyses. Since the results obtained by combining two 2D views, using STATIS method, are as satisfactory compared with a more heavy 3D landmark analysis, it was advantageous to use 2D, especially in the perspective of future analyses which could complement the present one by adding specimens using easily only photographs (lateral, dorsal), and not necessitating more heavy 3D scanning. In the present work, this also allowed us to make comparisons with, for instance, Pecsics et al.[8], also in 2D.

On dorsal view, 100 semi-landmarks were digitised to represent the contour of the braincase and orbits (as preserved in the fossil, i.e. with tympanic wings essentially missing). On lateral view, 4 landmarks (Supplementary Table 5) were digitised together with 65 semi-landmarks, also representing braincase (with 40 points) and orbits (with 25 points) contour (Supplementary Fig. 17).

Before placement of semi-landmarks in dorsal view, the fossil cranium was symmetrized (with better preserved parts of one side). In addition, for alignment of specimens in dorsal view, a semi-transparency mode of volume visualization was used with Avizo, so that the caudal end of foramen magnum was visible at the same distance to the caudal end of cranium, as the length (caudo-rostral) of the foramen magnum itself.

Before placement of landmarks and semi-landmarks in lateral view, skulls were aligned so that (as always in orthographic view) processi (such as tip of paroccipital process) were aligned (right and left ones superimposed).

Semi-landmarks are a special category, and are allowed to slide, in the analytical process, along the curve or outline to be captured and analysed, in order to minimize the bending energy necessary to produce the change in the outline relative to the reference form. Thereby, in the present analysis, semi-landmarks were treated as 'sliding landmarks', and allowed to slide as in Pecsics et al.[8] on owl skulls, using Tps Relw 32[7]. Then, the coordinates were transformed using the Procrustes superimposition method, with Tps Relw 32. The consensus configurations and relative warps were processed. The deformations across the owl taxa were visualised along PC1 and PC2 corresponding to these relative warps, using PAST 3[9]. Vectors of deformation and deformation grids helped visualise these deformations.

In an attempt to assess the contribution of characters caught by both views in the overall morphological variation of the assemblage, a STATIS compromise was realised using R, following Lavit et al. and Guenser et al.[10,11]. This analysis also allowed for the identification of correlation between characters quantified with the separate landmarks/semi-landmarks analyses.

PCA on measurements

For this PCA, all traditional measurements taken (on cranial and endocranial parts) were used (Supplementary Table 4) except measures of angles. Volumes and body mass were transformed as their cube root, and surfaces as their square root, in order to reduce too important scaling effects of these parameters (and their load relative to linear measurements). Then, the geometric mean (GM) of values in all these partly transformed measurements was calculated for every species. Finally, following the Mosiman's transform method[12], ln(value/MG) was calculated for every value in the table. This allows extraction of the non-directly size-dependent part of the shape variation. A PCA was realised on the resulting values.

A plot of the projection values of the specimens on PC1 (y) to specimens MG (x) was realised to evaluate the degree of allometry in variation across species, and to assess whether the fossil departs from such allometric trend (S.Fig. 7).

Ratios, bivariate and trivariate analyses

The PCAs results were completed by bivariate and trivariate plot analyses, as well as boxplot analyses (in particular for endocranial values), derived from the traditional measurements taken (Supplementary Table 4). These were potentially masked by the important effect of a relatively small skull and even smaller brain in *O. murivorus* (relative to body size), since the PCAs weighted all values with the Geometric Mean that includes body size variables. For instance, endocranial surface of selected parts of the brain (wulst W, optical lobe OL, cerebellum Crb, cerebral hemisphere) were plotted against the total endocranial surface; inner ear semi-circular canals lengths were plotted against cranium height; angles between two semi-circular canals were plotted against each other. These tests were carried out in order to detect particular ratios in extant species with particular ecology, and whether *O. murivorus* significantly differs in one way or another from *O. sunia*, and from the entire extant owls sample.

Supplementary Figures

Supplementary Fig. 1 First two principal components of the STATIS compromise based on the lateral and dorsal landmark/semi-landmark analyses and position of the specimens (**a**), and associated correlation circles showing both the projections of the two data sets (**b**) and the principal components of the separate PCAs (**c**). The first axis (horizontal) explains 39.8% of the total variance, and the second axis, 23.7%

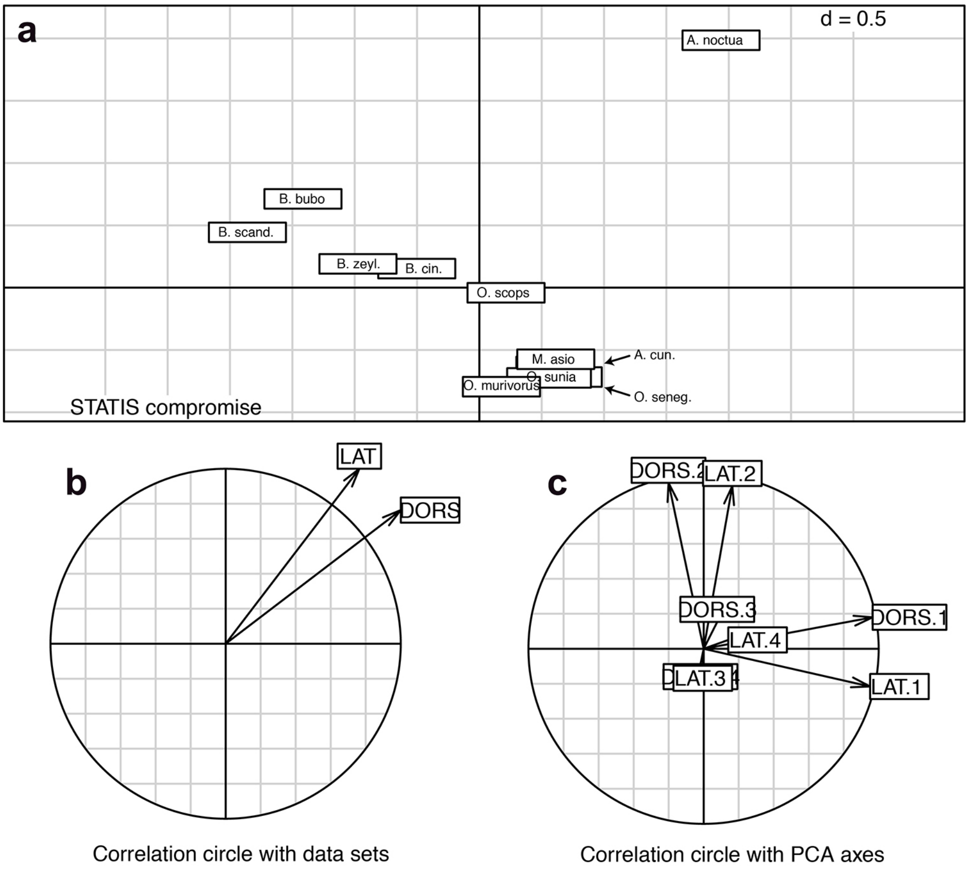

Supplementary Fig. 2 First two principal components (PC1 and PC2) of the PCA in landmark-semi-landmark analysis, in lateral view (relative warps). Deformations along PC1 and PC2 are illustrated (relative to 0)

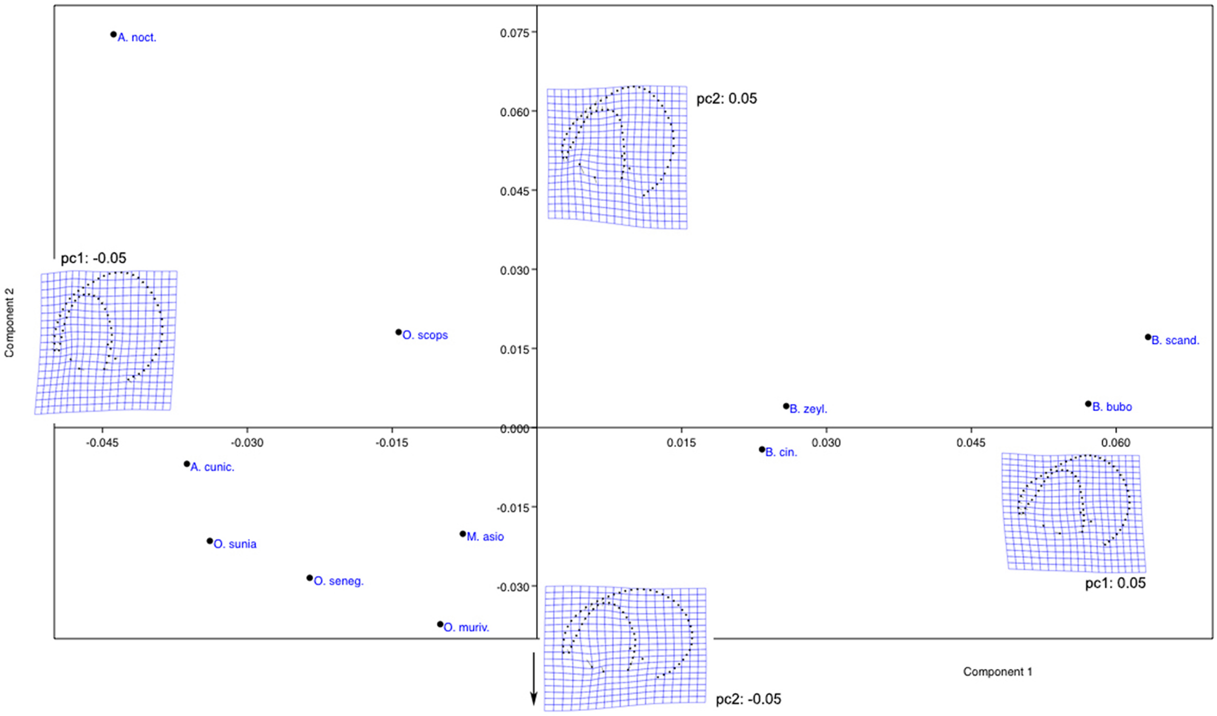

Supplementary Fig. 3 First two principal components (PC1 and PC2) of the PCA in semi-landmark analysis, in dorsal view (relative warps). Deformations along PC1 and PC2 are illustrated (relative to 0)

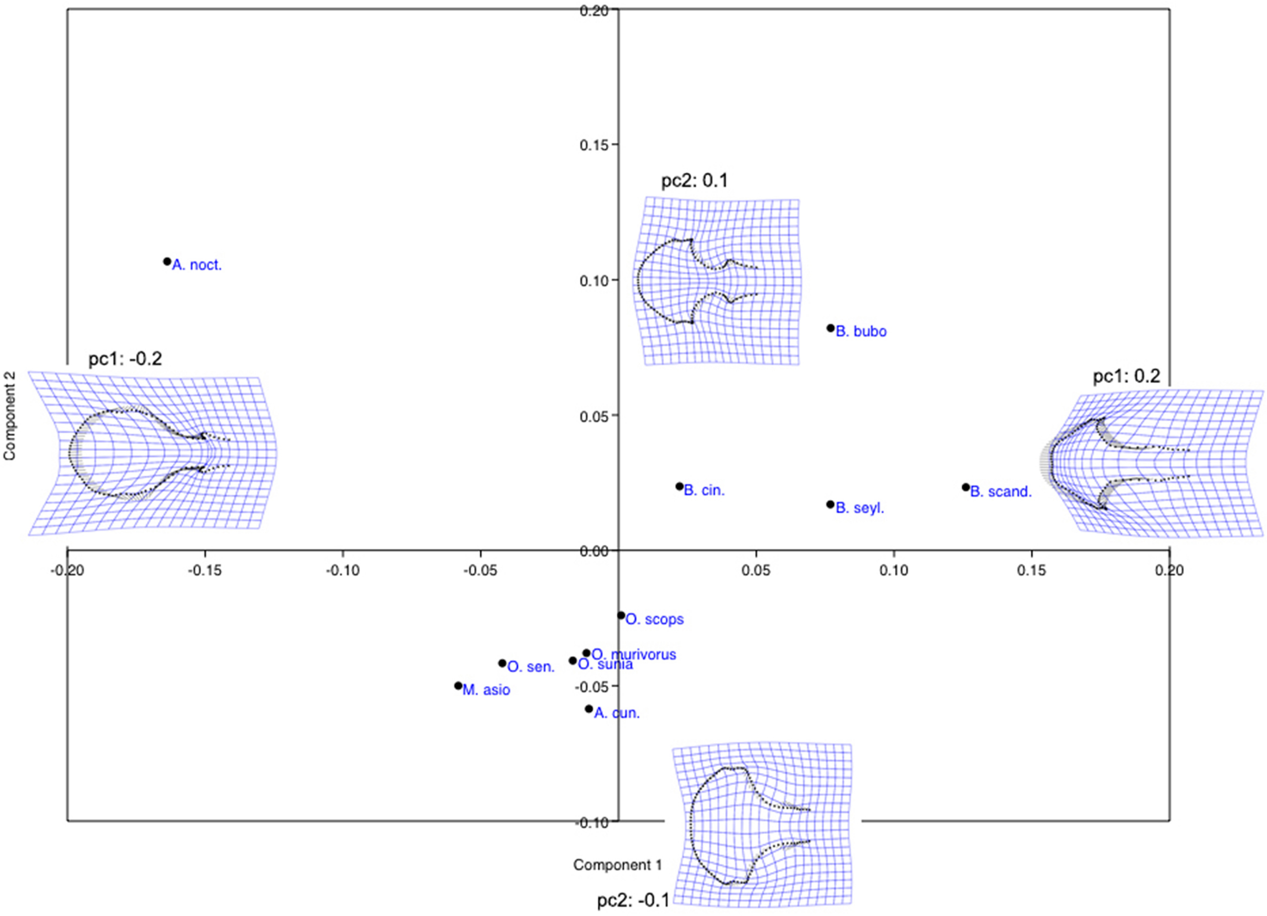

Supplementary Fig. 4 Deformation grid between *O. sunia* and *O. murivorus* in dorsal (**a**) and in lateral view (**b**). The sense of deformation is from tip of bar (*O. sunia*) to point (*O. murivorus*)

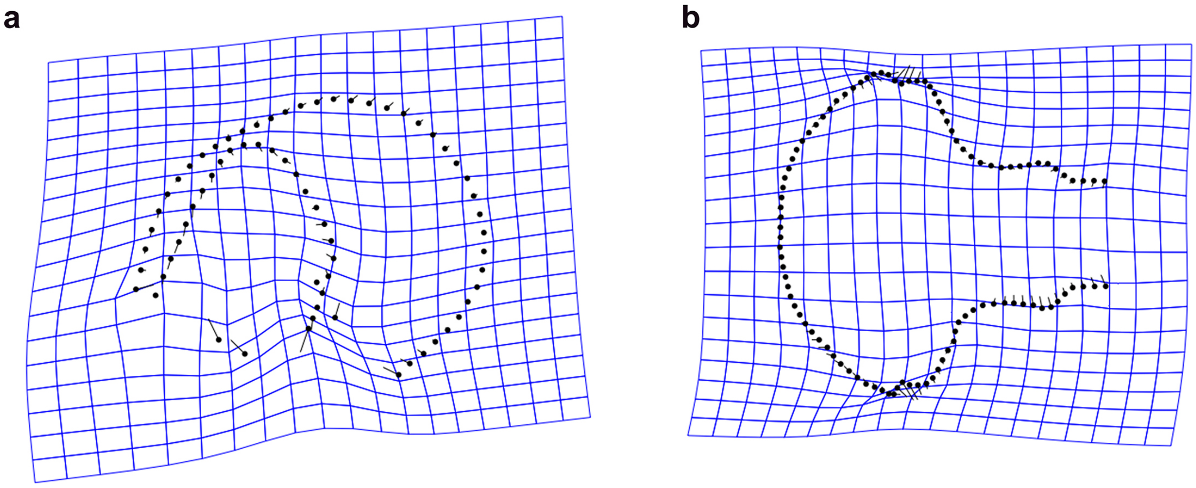

Supplementary Fig. 5 Scree plot of the eigenvalues of all the principal components (in blue), from the PCA of traditional measurements taken on crania and endocrania (except angles). The broken stick (in red) indicates that only the PC1 and PC2 must be retained for interpretation

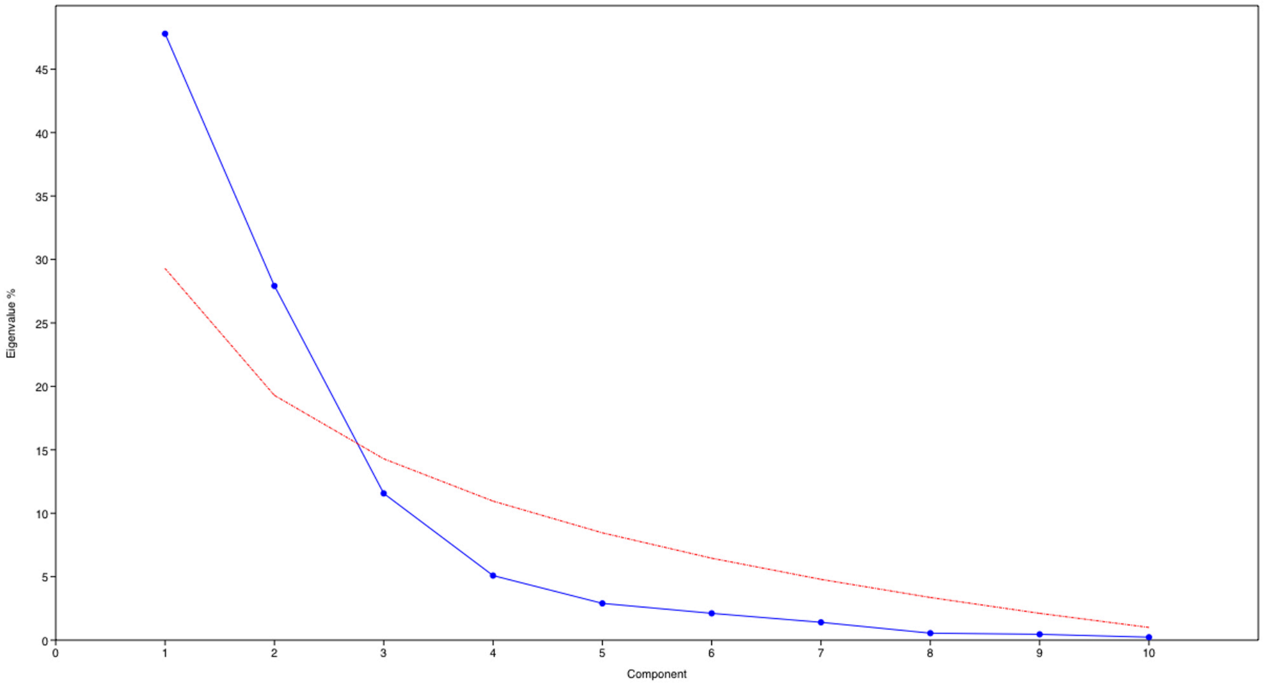

Supplementary Fig. 6 Result of the PCA based on traditional measurements (except angles). **a**, First two principal components (PC1 and PC2) of the PCA, showing specimens. **b**, First two principal components correlation circle, showing variables. The more diurnal extant species are underlined. Orange circle, *O. murivorus*; green-blue triangle, *O. sunia*

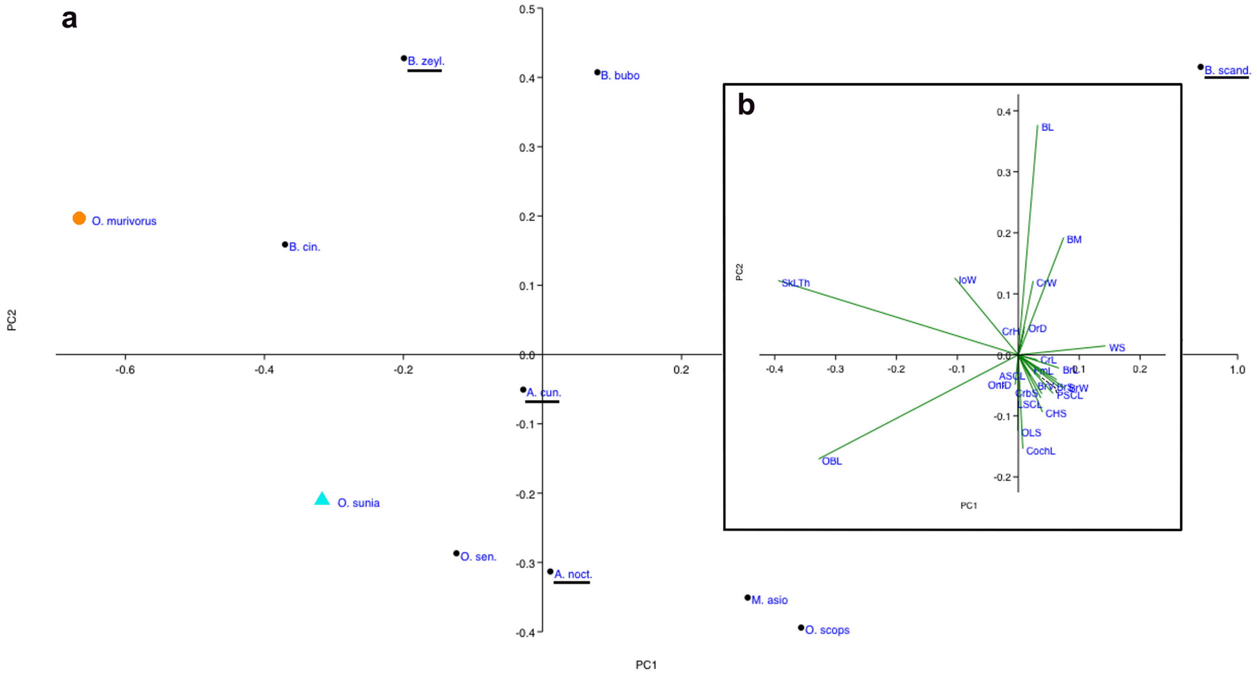

Supplementary Fig. 7 Plot of the specimens scores along PC1 (y) to their geometric means (x). The RMA regression r^2 is extremely low: 0.025. Large orange asterisk, *O. murivorus*; small green-blue asterisk, *O. sunia*

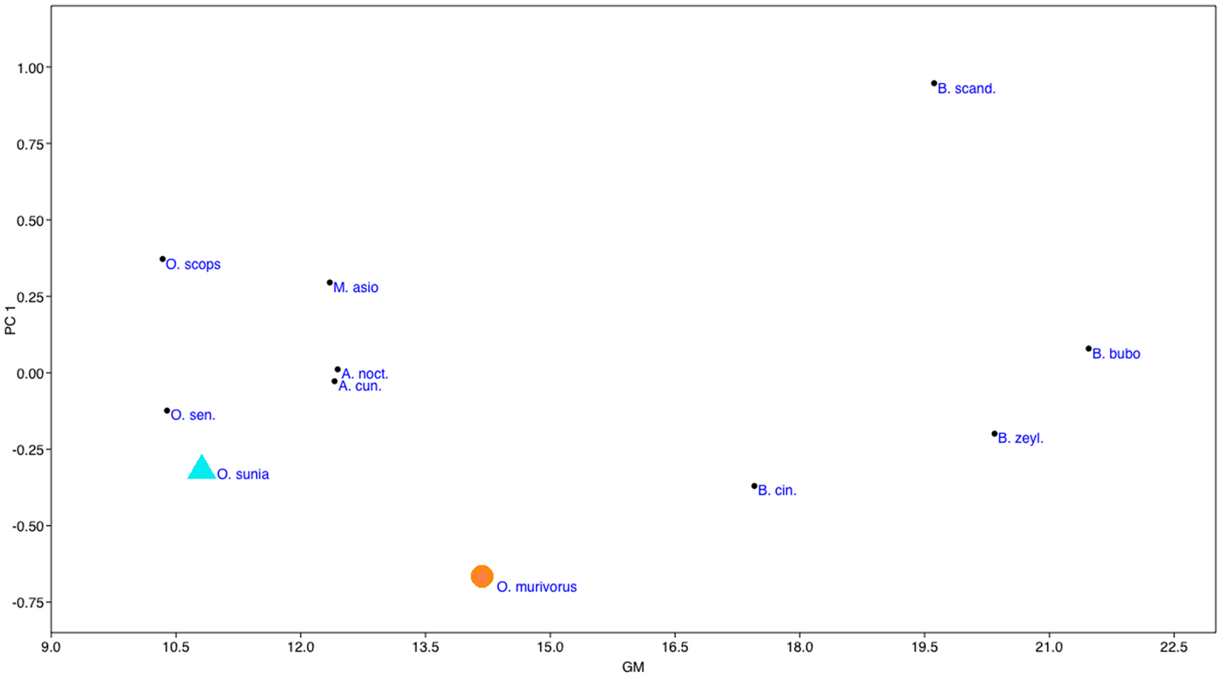

Supplementary Fig. 8 Plot of LOG cranium widths to LOG body lengths, with Y/X regression line (r^2 = 0.94). Slope = 0.7. The translucid grey zone represents a 95% confidence interval around the regression. Orange circle, *O. murivorus*; green-blue triangle, *O. sunia*, with dashed arrow symbolising evolutionary trajectory. *Otus murivorus* has the lowest cranium width ratio

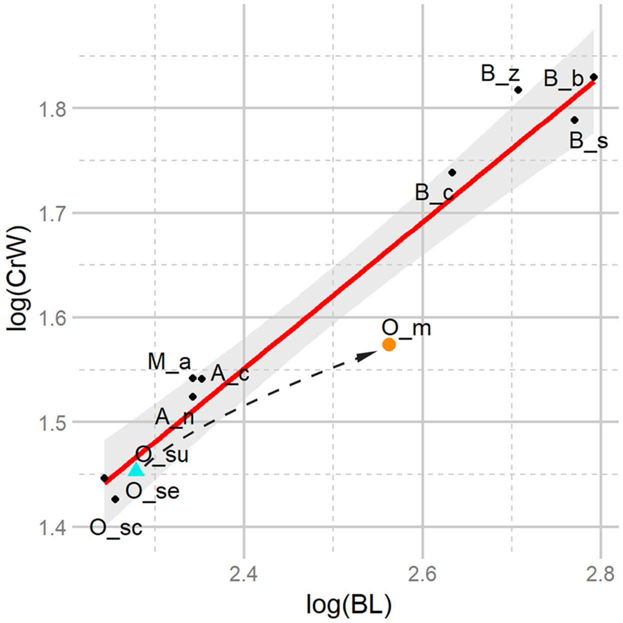

Supplementary Fig. 9 3D plot showing the distribution of the ratios of the lengths of the three semi-circular canals to cranium height, in the ten extant strigid species, and *O. murivorus* (in orange) which stands within the central group. *O. sunia* (in green-blue) exhibits the highest ratio values together with *O. scops*. In contrast, *Bubo zeylonensis* (in violet) has the lowest ratio values

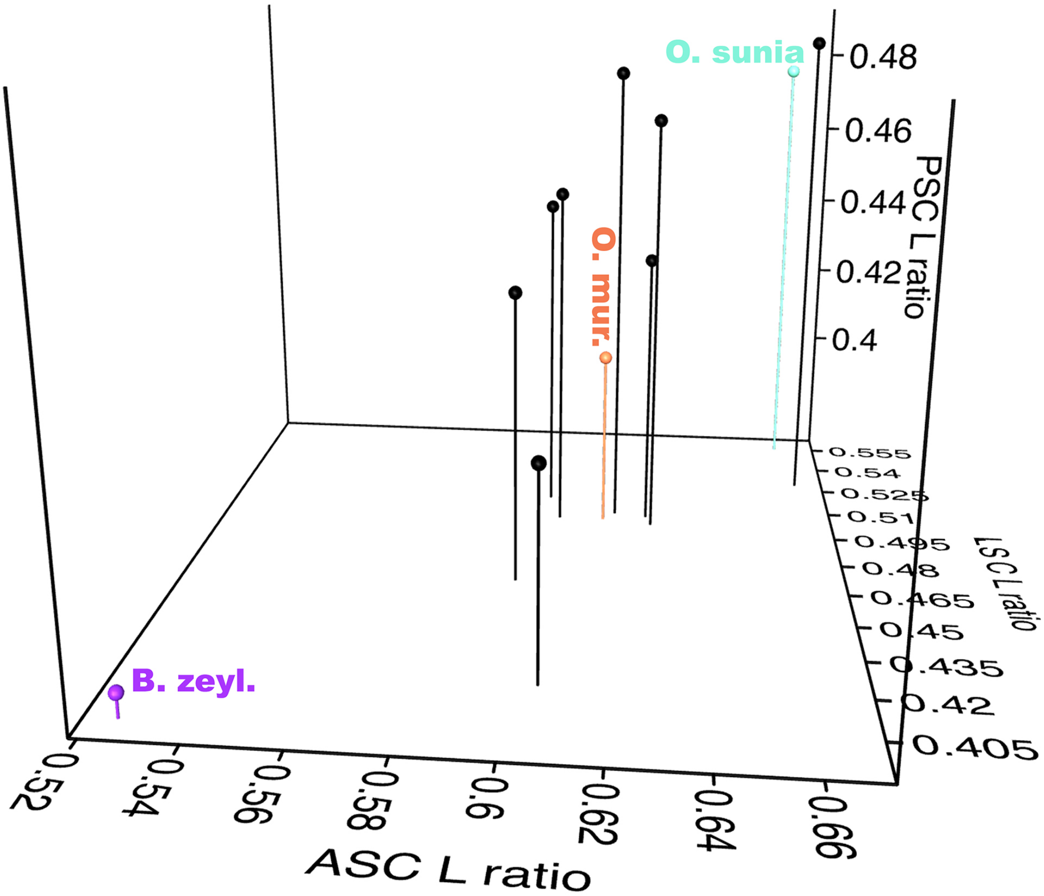

Supplementary Fig. 10 Angle of orbital margin convergence (OMC measured as in ref. [13]) to body length, in *O. murivorus* and a sample of extant strigid owls; with data from the present study combined with published data[13]. In orange, *O. murivorus*; in black, nocturnal and crepuscular species, including *O. sunia* in green-blue; in grey, the more diurnal (and crepuscular) species. Mean value for the more nocturnal species, 37.70°; for the more diurnal species, 35.64°

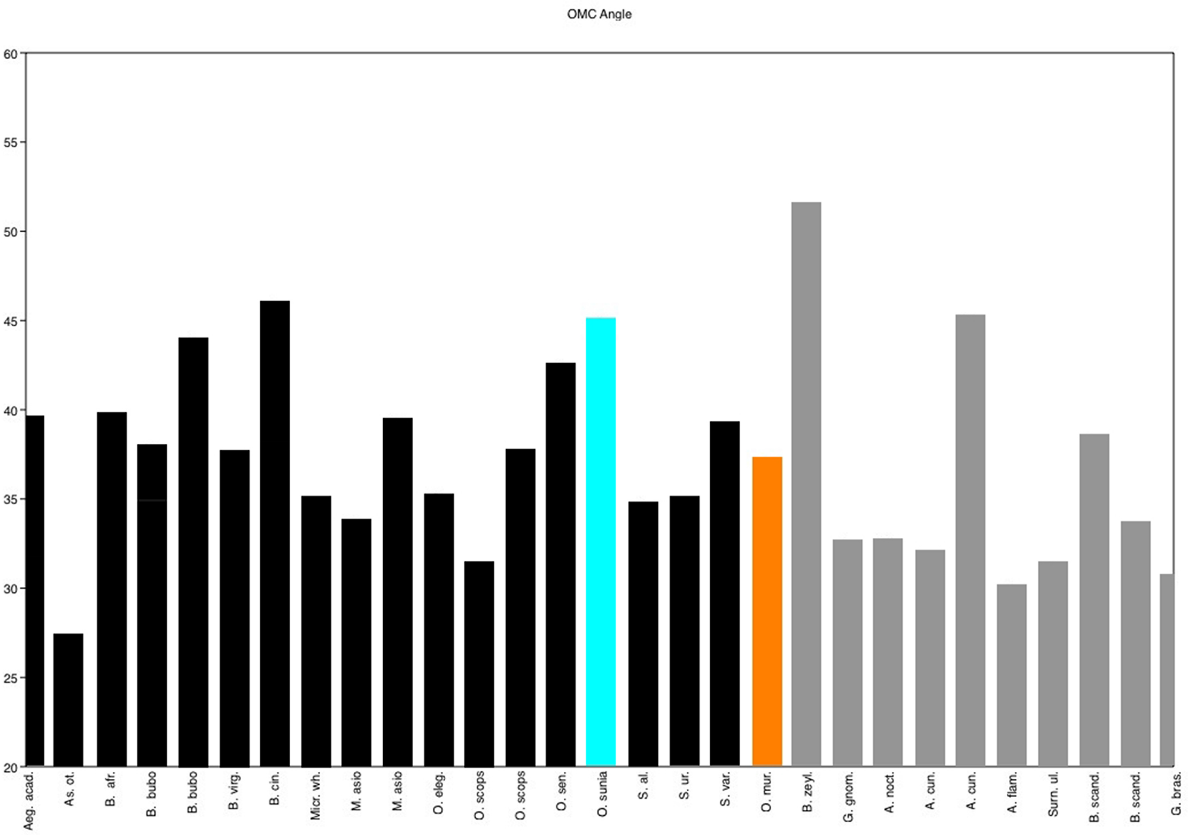

Supplementary Fig. 11 Scatter plot of posture angle (angle between foramen magnum plane and naso-frontal hinge) to body length. Orange circle, *O. murivorus*; green-blue triangle, *O. sunia*. RMA regression line in red (r^2=0.77)

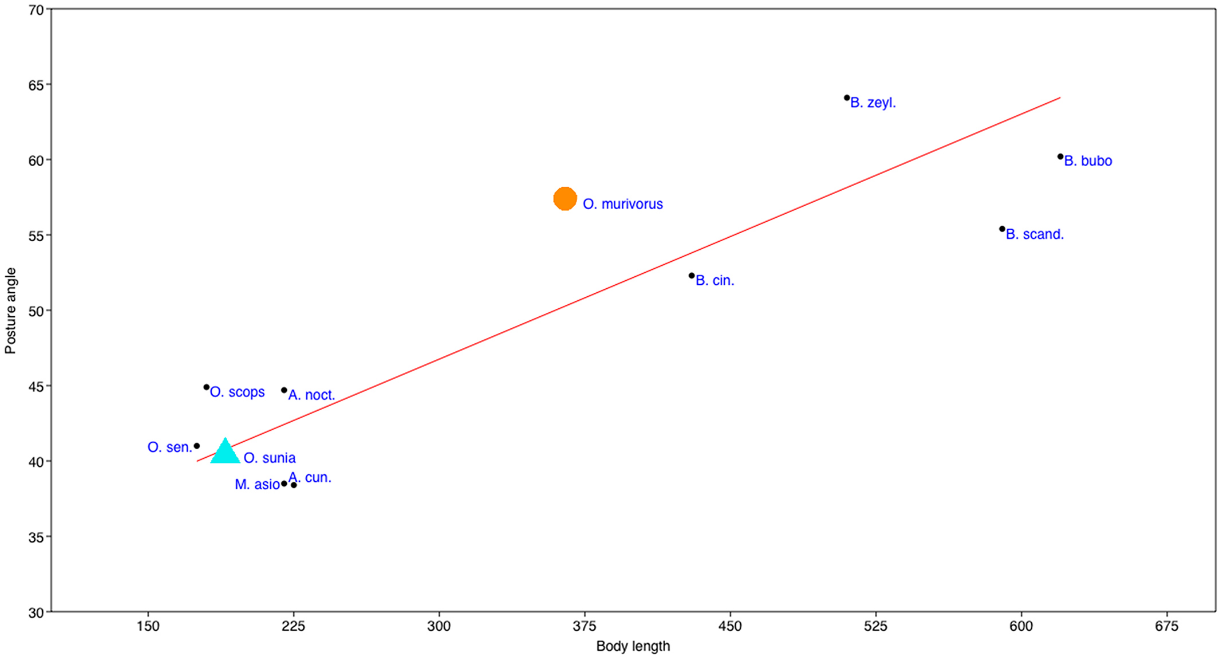

Supplementary Fig. 12 Measurements of distances and angles as taken on cranium (example of *A. cunicularia*, abbreviations refer to Supplementary Table 4) in lateral right (**a**) and dorsal (**b**) view. *OrD is taken using Avizo as a 3D length; other lengths were taken as 2D lengths. Scale bars, 20 mm

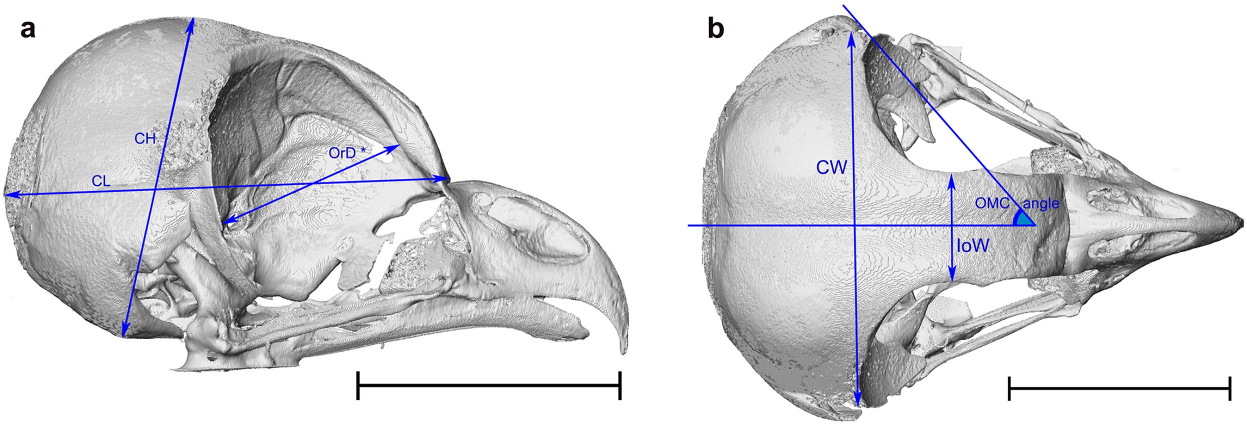

Supplementary Fig. 13 Truncated volume (sagittally) of *A. cunicularia*, showing how the posture angle was measured, as well as foramen magnum length (FmL). Scale bar, 10 mm

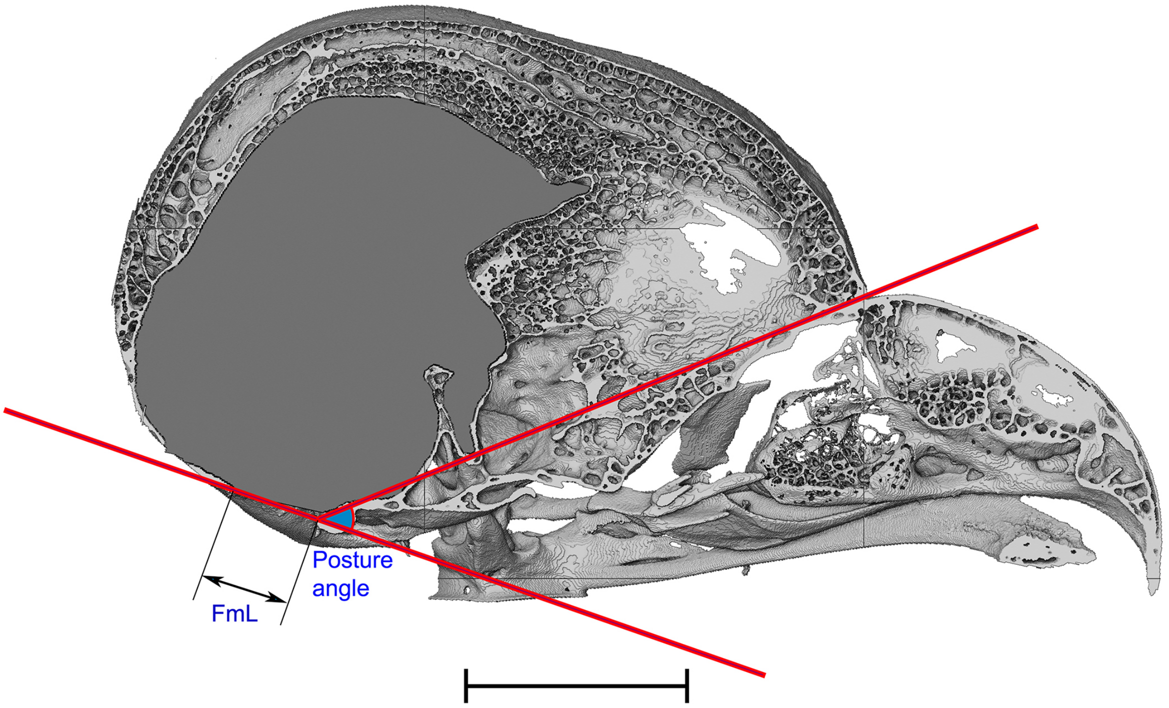

Supplementary Fig. 14 Volume rendering of *A. cunicularia*'s endocast in caudal (**a**) and lateral (**b**) views, showing endocast maximum width (BrW) and maximum length (BrL)

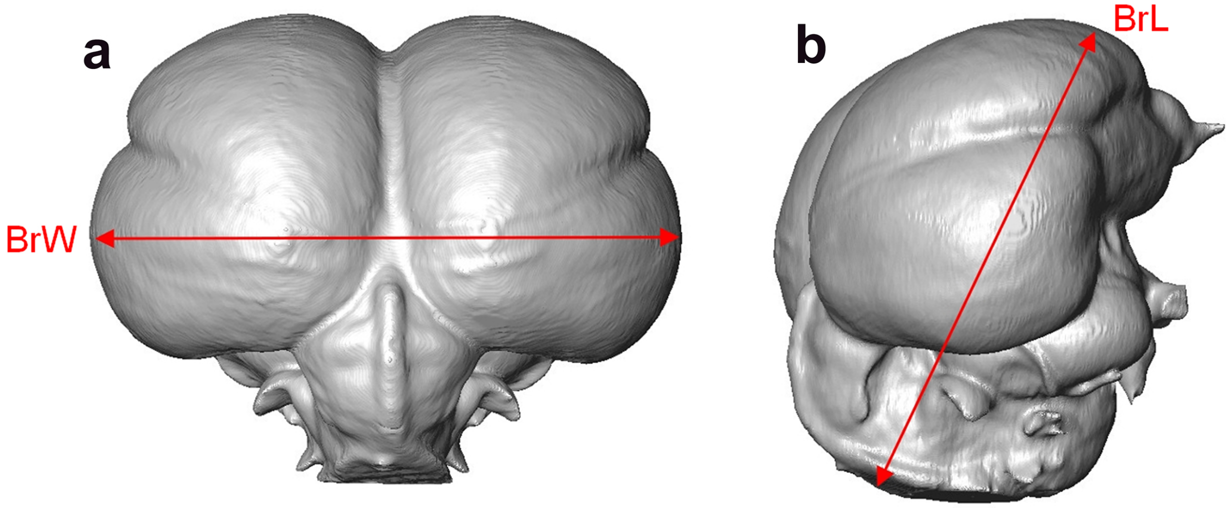

Supplementary Fig. 15 3D reconstruction of the avian left labyrinth (example of *A. cunicularia*) showing the three semicircular canals (**a**) and the process of angular measures (**b**, **c** and **d**). In blue: the anterior semicircular canal (ASC), in green: the posterior semicircular canal (PSC), in yellow: the lateral semicircular canal (LSC). The grey part includes the cochlear duct (CD). Alpha is the angle between LSC and PSC (**b**), beta between ASC and PSC (**c**), gamma between ASC and LSC

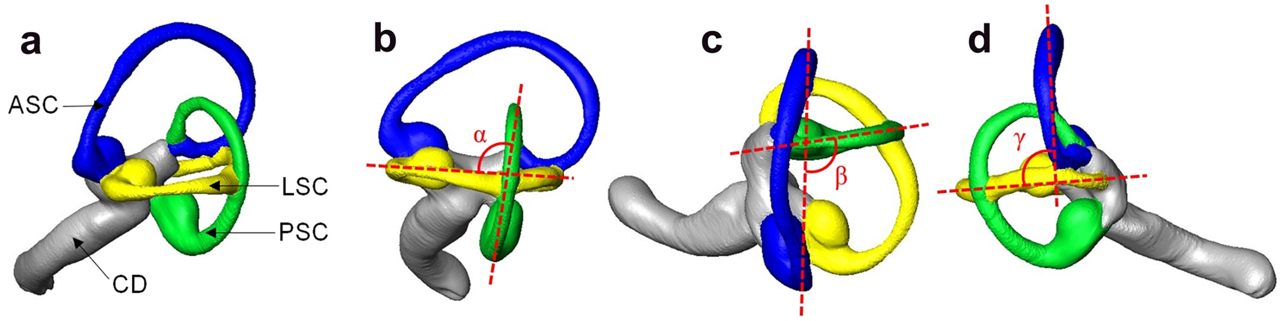

Supplementary Fig. 16 3D reconstruction of the avian cranial endocast (example of *A. cunicularia*). **a**, caudal view; **b**, left lateral view; **c**, left rostrolateral view; **d**, dorsal view. In blue: the wulst (w), in green: the cerebral hemisphere (cer), in orange: the cerebellum (cbl), in yellow: the optic lobe (opt). The red arrows show the olfactory bulb length (ob length) as measured. Scale bar, 10 mm

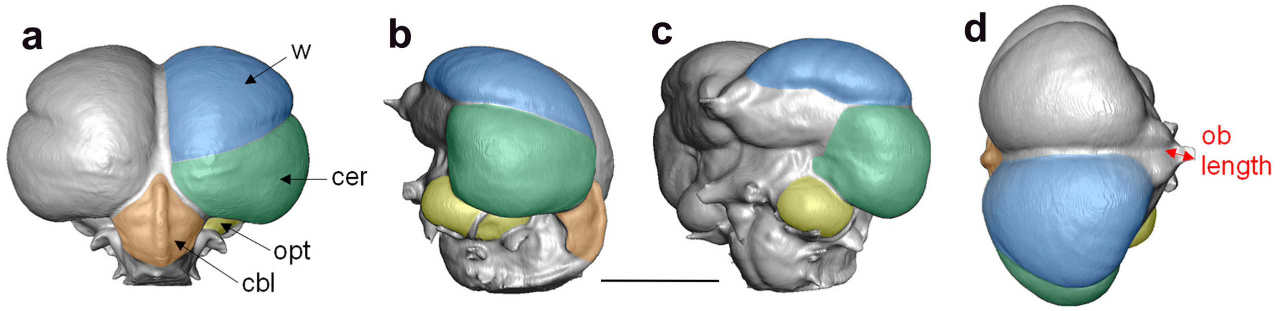

Supplementary Fig. 17 3D reconstruction of *A. cunicularia*'s skull showing landmarks (in red) and semi-landmarks (in blue) position, in dorsal (**a**) and lateral left (**b**) views. Description of landmarks associated with numbers is explained in Supplementary Table 5. Scale bars, 20 mm

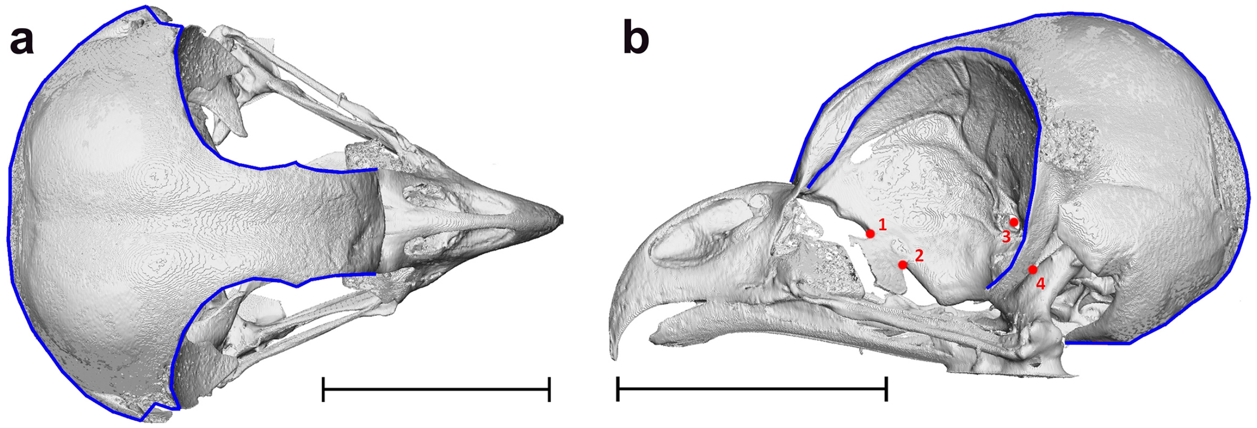

Supplementary Tables

PC	Eigenvalue	% variance
1	0.186618	47.788
2	0.109017	27.916
3	0.0451512	11.562
4	0.0198713	5.0885
5	0.011309	2.896
6	0.00824359	2.111
7	0.00547935	1.4031
8	0.00213734	0.54732
9	0.0017904	0.45848
10	0.000893751	0.22887

Supplementary Table 1 Eigenvalues and percentages of variance explained by the PCs after PCA on traditional measurements

	PC 1	PC 2	PC 3
A. cun.	-0.027661	-0.050634	0.54097
A. noct.	0.011298	-0.31308	0.10627
B. bubo	0.079032	0.40736	-0.047147
B. scand.	0.94686	0.41506	-0.031129
B. zeyl.	-0.19922	0.42759	0.097638
B. cin.	-0.37026	0.15883	-0.1626
M. asio	0.29516	-0.35073	-0.18643
O. scops	0.37211	-0.39404	-0.16696
O. sen.	-0.12376	-0.2868	0.013577
O. sunia	-0.31698	-0.21011	0.044612
O. murivorus	-0.66659	0.19655	-0.20881

Supplementary Table 2 Scores of individuals on PCs (PCA on traditional measurements)

	PC 1	PC 2	PC 3
CrL	0.054556	-0.014344	0.038712
CrH	0.016224	0.067404	0.0020131
CrW	0.042497	0.21042	0.0075113
IoW	-0.18127	0.21904	-0.1827
OrD	0.01758	0.079917	-0.037861
FmL	0.063386	-0.062983	-0.044356
SkLTh	-0.6856	0.21158	0.62255
OnfD	-0.0086462	-0.085138	-0.22467
BrV	0.11382	-0.089832	0.067812
BrS	0.11005	-0.071237	0.068176
BrL	0.11617	-0.037505	0.06859
BrW	0.13291	-0.095104	0.056112

WS	0.24884	0.025475	0.10164
CHS	0.069011	-0.16277	0.072983
OLS	-0.0015784	-0.21637	0.10477
CrbS	0.067362	-0.1106	0.13315
OBL	-0.57013	-0.29668	-0.60675
CochL	0.012834	-0.26741	0.11514
ASCL	0.033457	-0.060393	-0.030662
LSCL	0.064365	-0.12274	0.025976
PSCL	0.099179	-0.10988	-0.030676
BM	0.1298	0.33438	-0.05286
BL	0.055179	0.65477	-0.2746

Supplementary Table 3 Loadings of the variables on PCs

	Athene cunicularia	*Athene noctua*	*Bubo bubo*	*Bubo scandiacus*	*Bubo zeylonensis*	*Bubo cinerascens*	*Megascops asio*	*Otus scops*	*Otus senegalensis*	*Otus sunia*	*Otus murivorus*
CrL (CRANIUM LENGTH)	34.35	35.99	58.06	56.22	54.08	46.17	34.77	27.74	27.78	28.47	37.43
CrH (CRANIUM HEIGHT)	24.66	24.78	42.5	41.98	42.83	36.43	24.21	20.08	21.16	20.99	28.26
CrW (CRANIUM WIDTH)	34.77	33.42	67.57	61.41	65.62	54.74	34.8	26.68	27.95	28.39	37.5
IoW (MIN. INTERORBITAL WIDTH OF FRONTALS; DORSAL VIEW)	9.98	8.56	18.13	17.64	24.56	16.68	9.92	9.13	9.96	11.45	16.03
OrD (ORBIT DIAMETER)	18.21	17.62	33.39	29.67	34.3	27.83	19.57	15.29	15.44	15.32	20.1
FmL (FORAMEN MAGNUM LENGTH)	4.16	4.81	7.53	7.22	7.86	6.54	4.4	4.26	3.74	4.06	4.45
SkLTh-W (THICKNESS OF CRANIUM BONE WALL; MIN. IN WULST REGION)	2.04	1.75	0.91	1.5	2.87	2.93	1.71	0.28	1.79	2.11	3.72
SkLTh (THICKNESS OF CRANIUM BONE WALL; MIN. IN CEREBRAL HEMISPHERE REGION)	2.98	2.17	3.84	1.86	4.53	3.59	1.4	1.14	1.83	2.26	3.49
OMC (ANGLE OF ORBITAL MARGIN CONVERGENCE)	45	34.5	43.8	38.3	51.3	45.8	39.3	37.5	42.4	44.9	37
POSTURE ANGLE (ANGLE OF THE FORAMEN MAGNUM PLANE TO NASO-FRONTAL HINGE IN LATERAL VIEW)	38.4	44.7	60.2	55.4	64.1	52.3	38.5	44.9	41	40.5	57.4
BrV (BRAIN VOLUME; MM3)	3929.9	4044.02	16605.38	17528.85	12843.6	8040.66	3874.6	2515.37	2213.69	2265.9	3880.41
BrS (BRAIN SURFACE; MM2)	1550	1560	4101	4216	3496	2538	1538	1121	1036	1064	1542
BrL (ENDOCAST MAX. LENGTH)	21.59	21.57	36.48	36.13	33.8	28.58	20.8	19.04	17.83	17.88	21.12
BrW (ENDOCAST MAX. WIDTH)	25.82	26.13	42.38	43.29	38.74	33.3	27.03	22.2	21.29	21.9	25.04
WS (WULST SURFACE)	223	180	705	706	500	302	217	171	121	122	170
CHS (CEREBRAL HEMISPHERE SURFACE)	204	234	467	493	456	352	213	150	146	149	205
OLS (OPTIC LOBE SURFACE)	49	42	93	81	89	74	48	31	32	30	47
CrbS (CEREBELLUM SURFACE)	56	61	134	128	104	82	49	34	37	33	57
OBL (OLFACTORY BULB LENGTH (AS BANG & COBB [1]))	2.04	2.9	4.21	2.2	3.9	5.1	2.9	2.19	2.5	2.78	4.84

CochL (COCHLEAR DUCT LENGTH)	7.13	6.54	9.57	8.81	9.93	7.73	7.56	5.92	5.29	5.86	7.03
SINUOSITY LSC	very sinuous (angular)	very sinuous (more sigmoid)	moderately sinuous	very sinuous	very sinuous	moderately sinuous	slightly sinuous	flat	moderately(-) sinuous	flat	Moderately(-) sinuous
THICKNESS CANALS	medium	+	medium (-)	medium	medium	-	+	-	medium	(medium) (-)	medium (+)
ANGLE LSC/ASC	99.6	102.6	96.8	92	101.1	99.6	99.2	100.3	102.7	99.8	100.5
ANGLE ASC/PSC	98.4	109	107.8	114.7	109.1	102.4	104.9	105	104.2	109.5	97.3
ANGLE PSC/LSC	90.3	90.6	90.3	84.7	93.7	91.8	90	94.7	92.4	91	88.9
ASCL (LENGTH ASC)	15.44	15.32	25.35	25.37	22.5	22.06	15.19	13.37	12.72	13.9	17.39
LSCL (LENGTH LSC)	12.44	12.54	19.79	21.08	17.09	15.27	12.1	10.62	10.89	11.65	14.19
PSCL (LENGTH PSC)	10.76	11.93	18.62	19.01	16.03	15.19	11.41	9.77	9.46	10	11.64
BM (BODY MASS (G))	195	170	1900	1730	1105	500	210	82	73	85	372
BL (BODY LENGTH)	225	220	620	590	510	430	220	180	175	190	365
CHL (CEREBR. HEMISPH. LENGTH)	14.81	15.39	23.9	22.98	21.15	18.7	15.11	12.67	12.01	13.07	14.8
BASICRANIAL AXIS LENGTH	9.58	9.9	13.6	12.7-13.7	13.8	11.4	7.81	7.2	7.7	7.26	11
MAX DIAMETER OPTICAL NERVE FORAMEN	1.46	1.86	3.72	2.61	2.59	2.36	1.75	1.57	1.58	1.48	1.99
OnfD (OPTICAL NERVE FORAMEN MEAN DIAMETER)	1.415	1.67	3.15	2.38	2.59	2.175	1.685	1.52	1.52	1.425	1.915
RATIO OF MEAN DIAMETER OF OPTICAL NERVE FORAMEN TO CRANIUM HEIGHT	*0.057*	*0.067*	*0.074*	*0.057*	*0.060*	*0.060*	*0.070*	*0.076*	*0.072*	*0.068*	*0.068*
LENGTH (CRANIO-CAUDAL) OF MAXILLO-MANDIBULAR FORAMEN FOR TRIGEMINAL NERVE $V_{2\text{-}3}$	1.01	1.60	1.62	1.44	1.30	1.14	0.80	0.82	1.01	0.89	0.93
HEIGHT (DORSO-VENTRAL) OF MAXILLO-MANDIBULAR FORAMEN FOR TRIGEMINAL NERVE $V_{2\text{-}3}$	0.76	1.05	1.74	1.38	1.28	1.11	0.56	0.67	0.93	0.60	0.62
RATIO OF MEAN DIAMETER OF MAXILLO-MANDIBULAR FORAMEN TO CRANIUM HEIGHT	*0.036*	*0.053*	*0.040*	*0.034*	*0.030*	*0.031*	*0.028*	*0.037*	*0.046*	*0.035*	*0.027*

Supplementary Table 4 Traditional measurements and other observations, undertaken on *O. murivorus* and comparative extant specimens. Linear measurements are in mm, surfaces in mm^2, volumes in mm^3, and angles in degrees. NB: The estimation of body mass for *O. murivorus* was derived as follows. Using the regression equation "Predatory birds" of Campbell and Marcus[14] with the minimum femur shaft width as the best body mass proxy, a mean minimum femur shaft width of 4.71 mm for *O. murivorus* (after refs. [2,15]) yielded a mean body mass of 372 g. The mean body length of 365 mm was estimated by Louchart et al.[1]

Landmark n°	Description
**1**	Rostral end of the left palatines junction with mesethmoid

	(interorbital septum)
**2**	Caudal end of the left palatines junction with mesethmoid (interorbital septum)
**3**	Left optic nerve foramen
**4**	Extremity of the left zygomatic process

Supplementary Table 5 Description of landmarks placed on the lateral left view of the skulls under study (cf Supplementary Fig. 17)

References

1. Louchart, A. *et al.* Ancient DNA reveals the origins, colonization histories, and evolutionary pathways of two recently extinct species of giant scops owl from Mauritius and Rodrigues Islands (Mascarene Islands, south-western Indian Ocean). *J. Biogeogr.* 45, 2678–2689 (2018).
2. Günther, A. & Newton, E. The extinct birds of Rodriguez. *Philosophical Transactions of the Royal Society of London* **168**, 423–437, pl. XLI–XLIII (1879).
3. Cheke, A. S. & Hume, J. P. *Lost Land of the Dodo: the Ecological History of the Mascarene Islands* (A and C Black, London, 2008).
4. Milne-Edwards, A. Recherches sur la faune ancienne des Iles Mascareignes. *Annales des Sciences Naturelles—Zoologie et Paléontologie* **5** (19), 1–31, plates 11–15, (1874).
5. Bang, B. G. & Cobb, S. The size of the olfactory bulb in 108 species of birds. *Auk* **85**, 55–61 (1968).
6. Torres, C. R. & Clarke, J. A. Nocturnal giants: evolution of the sensory ecology in elephant birds and other palaeognaths inferred from digital brain reconstructions. *Proc. R. Soc. B* **285**, 20181540 (2018).
7. Rohlf, F. J. *TpsDig, version 2.16* (Department of Ecology and Evolution, State University of New York, Stony Brook, USA 2010).
8. Pecsics, T., Laczi, M., Nagy, G., Kondor, T. & Csörgö, T. Analysis of skull morphometric characters in owls (Strigiformes). *Ornis Hungarica* **26**, 41–53 (2018).
9. Hammer, Ø., Harper, D. A. T. & Ryan, P. D. PAST: paleontological statistics software package for education and data analysis. *Palaeontologia Electronica* **4**, 1–9 (2001).
10. Lavit, C., Escoufier, Y., Sabatier, R. & Traissac, P. The ACT (STATIS method). *Computational Statistics & Data Analysis* **18**, 97–119 (1994).
11. Guenser, P. *et al.* Deciphering the roles of environment and development in the evolution of a Late Triassic assemblage of conodont elements. *Paleobiology* **45**, 440–457 (2019).
12. Darroch, J. N. & Mosiman, J. N. Canonical and principal components of shape. *Biometrika* **72**, 241–252 (1985).
13. Menegaz, R. A. & Kirk, E. C. Septa and processes: convergent evolution of the orbit in haplorhine primates and strigiform birds. *J. Hum. Evol.* **57**, 672–687 (2009).
14. Campbell, K. E. Jr & Marcus, L. The relationship of hindlimb bone dimensions to body weight in birds. *Natural History Museum of Los Angeles County, Science Series* **36**, 395–412 (1992).
15. Mourer-Chauviré, C., Bour, R., Moutou, F. & Ribes, S. *Mascarenotus* nov. gen. (Aves, Strigiformes), genre endémique éteint des Mascareignes et *M. grucheti* n. sp., espèce éteinte de la Réunion. *C. R. Acad. Sci. Paris, sér. II* **318**, 1699–1706 (1994).
